# Thermal optima in the hypoxia tolerance of marine ectotherms: Physiological causes and biogeographic consequences

**DOI:** 10.1371/journal.pbio.3002443

**Published:** 2024-01-16

**Authors:** Martin-Georg A. Endress, Justin L. Penn, Thomas H. Boag, Benjamin P. Burford, Erik A. Sperling, Curtis A. Deutsch

**Affiliations:** 1 School of Oceanography, University of Washington, Seattle, Washington, United States of America; 2 Institute of Zoology, University of Cologne, Cologne, Germany; 3 Department of Geosciences, Princeton University, Princeton, New Jersey, United States of America; 4 Institute of Marine Sciences, University of California Santa Cruz, Santa Cruz, California, United States of America; 5 National Oceanic and Atmospheric Administration, National Marine Fisheries Service, Southwest Fisheries Science Center, Santa Cruz, California, United States of America; 6 Department of Earth and Planetary Sciences, Stanford University, Stanford, California, United States of America; 7 High Meadows Environmental Institute, Princeton University, Princeton, New Jersey, United States of America; Imperial College of Science Technology and Medicine: Imperial College London, UNITED KINGDOM

## Abstract

The minimum O_2_ needed to fuel the demand of aquatic animals is commonly observed to increase with temperature, driven by accelerating metabolism. However, recent measurements of critical O_2_ thresholds (“*P*_*crit*_”) reveal more complex patterns, including those with a minimum at an intermediate thermal “optimum”. To discern the prevalence, physiological drivers, and biogeographic manifestations of such curves, we analyze new experimental and biogeographic data using a general dynamic model of aquatic water breathers. The model simulates the transfer of oxygen from ambient water through a boundary layer and into animal tissues driven by temperature-dependent rates of metabolism, diffusive gas exchange, and ventilatory and circulatory systems with O_2_-protein binding. We find that a thermal optimum in *P*_*crit*_ can arise even when all physiological rates increase steadily with temperature. This occurs when O_2_ supply at low temperatures is limited by a process that is more temperature sensitive than metabolism, but becomes limited by a less sensitive process at warmer temperatures. Analysis of published species respiratory traits suggests that this scenario is not uncommon in marine biota, with ventilation and circulation limiting supply under cold conditions and diffusion limiting supply at high temperatures. Using occurrence data, we show that species with these physiological traits inhabit lowest O_2_ waters near the optimal temperature for hypoxia tolerance and are restricted to higher O_2_ at temperatures above and below this optimum. Our results imply that hypoxia tolerance can decline under both cold and warm conditions and thus may influence both poleward and equatorward species range limits.

## Abstract 2

The minimum oxygen level needed to fuel the demand of aquatic animals is commonly observed to increase with temperature, driven by accelerating metabolism. However, this study uses species measurements and a dynamical model to reveal how oxygen supply can reduce hypoxia tolerance and limit biogeographic ranges of marine ectotherms under both cold and warm conditions.

## Introduction

Climate change is raising temperatures throughout the upper ocean, while decreasing its oxygen content. These trends are among the most robustly observed and well-understood aspects of global ocean change [1]. They also pose a major challenge for marine ectotherms, whose metabolic rates rise exponentially with temperature [2,3], requiring a concomitant increase in O_2_ supply to maintain aerobic energy balance that is at odds with the ocean’s declining global O_2_ inventory [4,5]. The temperature-dependent hypoxia tolerance of marine species already limits their geographic distributions, most commonly at the equatorward (warm) and/or deep (low O_2_) range edge of species distributions [6–9], yielding a simple physiological mechanism for understanding species responses to climate change [10,11].

The minimum environmental O_2_ at which an organism can sustain its resting metabolism is typically reported as a critical pressure (*P*_*crit*_) and remains the most common measure of hypoxia tolerance, despite challenges associated with its experimental determination and interpretation [12–15]. In most studied species, *P*_*crit*_ increases with temperature, implying that their O_2_ demand accelerates faster with warming than their rate of O_2_ supply [6]. Some species show a decrease in *P*_*crit*_ as temperatures rise, implying that supply accelerates faster than demand, although this has rarely been observed [16,17]. In recent experiments with higher than typical sample size over a broad temperature range, species exhibited both a decline in *P*_*crit*_ as temperatures rise from the coldest water, followed by an increase from further warming, resulting in a minimum *P*_*crit*_, and thus a maximum hypoxia tolerance, at an intermediate optimum temperature [18,19]. While the individual processes of supply and demand all tend to increase steadily with temperature [20,21], these bowl-shaped *P*_*crit*_ curves require that the ratio of these rates exhibits a more complex relationship to temperature.

Thermal optima for hypoxia tolerance have been posited [22,23], but empirical support has been limited, in part because *P*_*crit*_ is commonly estimated at too few temperatures, typically focused at the warm end of species tolerance ranges most relevant to anthropogenic climate change. These data limitations prevent the development of mechanistic and quantitative models capable of evaluating the role of hypoxia tolerance at the cold edge of range limits, and the associated implications of climate change, especially for populations not living near a species’ warm range limit, or exposed to ocean cooling.

To examine the prevalence of thermal optima in hypoxia tolerance, diagnose the physiological conditions under which it can arise, and evaluate its relevance to species biogeography, we combined new laboratory experiments from species across multiple phyla, a dynamic model of O_2_ supply in aquatic ectotherms, and species biogeographic distribution data. Among all studied species, we find complex *P*_*crit*_ behavior across a broad range of temperatures. A general model of aquatic water breathers demonstrates the conditions under which thermal optima can emerge from the multistep nature of the O_2_ supply chain, and analysis of published laboratory data suggests that marine species commonly meet those conditions.

The behavior of the dynamic model can be reproduced with a generalized model of temperature-dependent O_2_ supply to demand ratios, termed “the Metabolic Index” [6], which captures a wide range of observed *P*_*crit*_ curves. Finally, we present evidence that *P*_*crit*_ curves with a thermal optimum are also reflected in the biogeography of marine species and thus may explain the cold limit in such species geographic ranges.

## Results

### Laboratory observations

**Fig 1:**
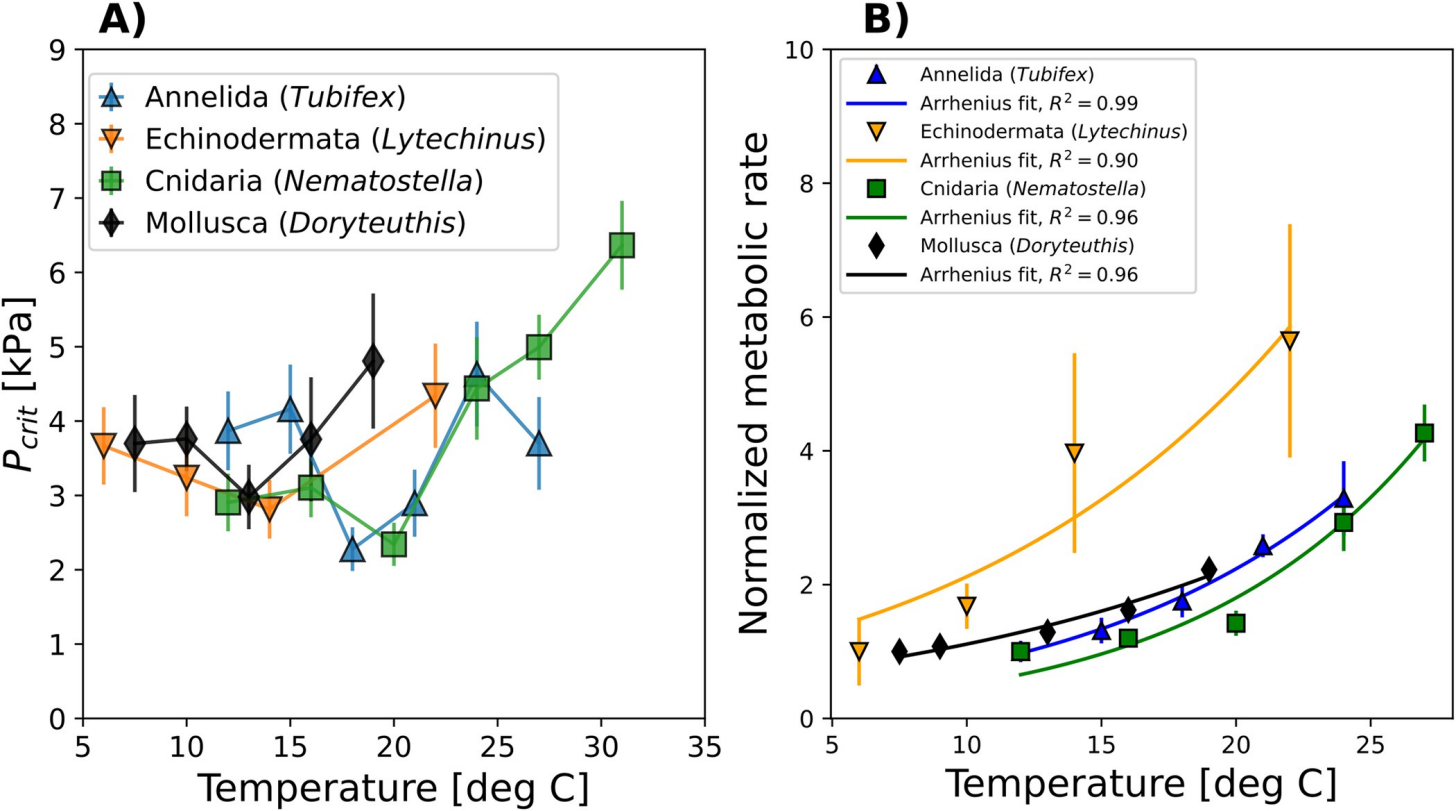
Temperature-dependent critical oxygen pressures (*P*_*crit*_) of 4 marine invertebrate species display thermal optima even as metabolic demands increase exponentially. **(A)** New closed system respirometry experiments exhibit a minimum of *P*_*crit*_ (mean ± SE) at intermediate temperatures, indicating a thermal optimum in hypoxia tolerance. The species include an oligochaete worm (*Tubifex tubifex*), a sea urchin (*Lytechinus pictus*), an anemone (*Nematostella vectensis*), and a cephalopod (*Doryteuthis opalescens*). (**B)** Normalized mass-specific metabolic O_2_ demand (mean ± SD) follows a simple Arrhenius relationship in all species and cannot explain the complex patterns observed in *P*_*crit*_. Metabolic rate data are plotted relative to the rate at the coldest measured temperature within a species. See text for details. The data underlying this figure can be found in S1 Data. Untreated experimental data can be found in https://doi.org/10.6084/m9.figshare.24236257.v1.

To evaluate the prevalence of nonexponential *P*_*crit*_ curves, we measured *P*_*crit*_ and metabolic rates across a wide temperature spectrum at high sample sizes for 4 previously unmeasured aquatic invertebrate species (Fig 1 and Fig A in S1 Text). These species span 4 different phyla, have multiple modes of oxygen supply, and regularly encounter temporal or spatial variability in environmental pO_2_. Following published respirometry protocols (Materials and methods), we conducted *P*_*crit*_ trials for the freshwater oligochaete worm *Tubifex tubifex* (*n* = 133), the outer shelf/upper slope sea urchin *Lytechinus pictus* (*n* = 39) from California, and the Atlantic intertidal anemone *Nematostella vectensis* (*n* = 107). We also used *P*_*crit*_ measurements of groups of the squid *Doryteuthis opalescens* (*n* = 14 groups of 15 to 30 individuals), which is exposed to strong gradients of temperature and O_2_ in the California Current System [24]. To robustly estimate mean species *P*_*crit*_ at each temperature, we performed the experiments on a much larger population of individuals (10s to >100) than in most previous studies. Full *P*_*crit*_ and metabolic rate data are provided in the Supporting information (Fig A in S1 Text and S1 Data). Unprocessed O_2_ drawdown curves can be found in https://doi.org/10.6084/m9.figshare.24236257.v1.

Measurements of all species reveal complex *P_crit_* patterns across the studied temperature range (Fig 1A) even as metabolic rates increase exponentially with warming (Fig 1B). Despite the considerable variability and uncertainty in individual *P*_*crit*_ estimates, the large number of measured individuals reveal a common pattern for all species. At intermediate temperatures, the mean *P*_*crit*_ across individuals is lower than at warmer and colder temperatures, indicating a thermal optimum in resting hypoxia tolerance. The location, depth, and width of this optimum varies across species. For example, *T*. *tubifex* shows a deep bowl with a significant minimum in *P*_*crit*_ at intermediate temperatures (Dunnett’s test, comparing the 2 intermediate temperatures to the 2 colder and warmer temperatures, *p* = 0.00039 and *p* = 0.048, respectively). *N*. *vectensis* features a weaker but significant drop in *P*_*crit*_ at 20°C relative to colder temperatures (*t* test, *p* = 0.031), followed by a sharp rise at warmer temperatures. The broad bowl shapes seen in *L*. *pictus* and *D*. *opalescens* include thermal minima in *P*_*crit*_ but are not statistically significant (Dunnett’s test, *p* > 0.05).

These new respirometry data combined with published data [18,19] indicate that thermal optima are found in multiple phyla and across multiple modes of oxygen supply (e.g., gills and a blood vascular system in squid versus cutaneous respiration in anemones) and may therefore represent a widespread pattern. These patterns cannot be explained by the measured temperature sensitivity of metabolic O_2_ demand alone, which rises throughout the temperature range (Fig 1B), thus implying a key role for O_2_ supply mechanisms.

### Dynamic model

**Fig 2:**
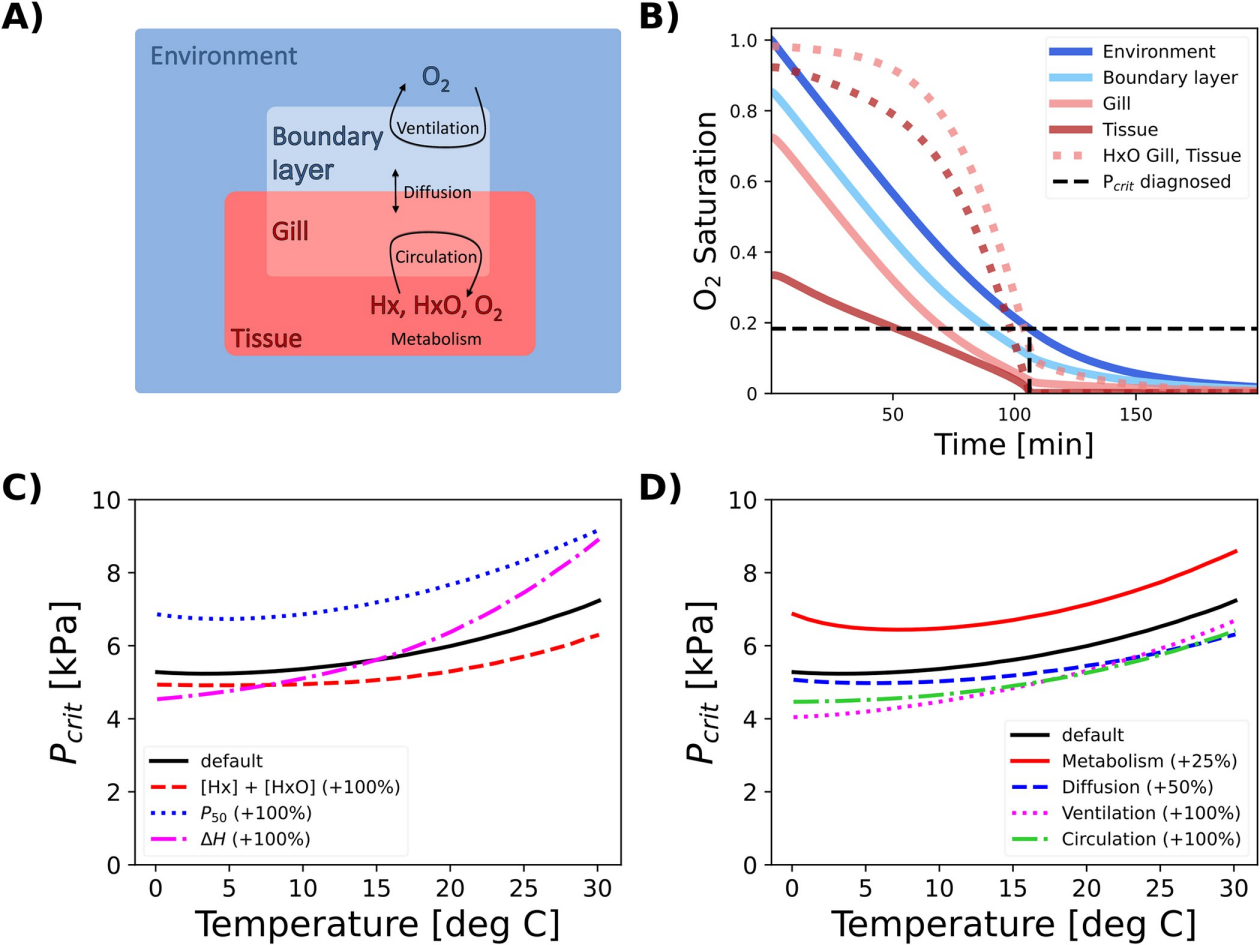
Overview of the model used to investigate the effects of a multistep O_2_ supply chain. **(A)** The model tracks the concentrations of O_2_ as well as unbound and bound O_2_ transporting proteins (“Hx,” “HxO”) in 4 compartments representing external and internal volumes of water or body fluid, which are connected through a linear O_2_ supply chain with external ventilation, diffusion, and internal circulation. **(B)** A model run at a single temperature resembles a closed system respirometry experiment. The saturation of O_2_ (solid colored) and proportion of HxO (dashed colored) decline in all compartments until the O_2_ level in the metabolizing tissue (dark red) reaches a critical limit near zero, at which point metabolic consumption slows down and *P*_*crit*_ (dashed black) can be determined from the rate of environmental O_2_ depletion. **(C)** Effects of increasing the concentration, half-saturation pressure (*P*_50_), or temperature sensitivity (Δ*H*) of O_2_ transport protein on the *P*_*crit*_ curve. **(D)** Effects of increasing the rate coefficients of biophysical supply and demand processes. A higher metabolism elevates the curve, while increasing the rate of any supply process lowers it. No experimental data underlie this figure. Code underlying this figure can be found in https://doi.org/10.6084/m9.figshare.24547255.

To explore the conditions that lead to a thermal optimum in hypoxia tolerance, we develop a dynamic model of O_2_ supply and demand in water-breathing animals. The model simulates the transfer of O_2_ from the environment to the metabolizing tissues of an organism across a range of temperatures using a system of coupled nonlinear ordinary differential equations (ODEs). To make the model generally applicable to aquatic animals, we include all the potential pools and fluxes of O_2_, including external ventilation of water from the ambient fluid to the boundary layer at the exchange surface, the molecular O_2_ diffusion across that surface, and internal flux of O_2_ to metabolizing body tissues, which may be mediated by a circulatory system (Fig 2A). In addition to dissolved O_2_, the model also tracks the concentration of the bound and unbound forms of an oxygen-transport protein such as hemoglobin or hemocyanin (denoted HxO and Hx, respectively), which bind and release molecular O_2_ according to the associated chemical equilibrium. This is captured by the pO_2_ at half-saturation (denoted *P*_50_) and the enthalpy (Δ*H*) of the binding reaction, which governs the temperature dependence of that equilibrium [25] (S1 Text).

Each of the 3 O_2_ supply processes (ventilation, diffusion, and circulation) is described by a rate *S*_*i*_ that is represented as the product of the pO_2_ difference between the respective compartments, Δ_*i*_*pO*_2_, and a temperature-dependent rate coefficient ${\hat{\alpha }}_{i}(T)$ that characterizes the kinetics of that process:

$$S_{i}(T)={\hat{\alpha }}_{i}(T)\cdot \Delta_{i}pO_{2}$$ (1)

The 3 rate coefficients—flow rates of ventilated water and circulated blood and the diffusivity of O_2_—each vary exponentially with temperature (Arrhenius function), as does the metabolic rate, but with distinct temperature sensitivities. The resulting 8 parameters (3 supply rate coefficients, the metabolic rate, and the temperature sensitivity of each) along with the 3 chemical parameters (*P*_50_, Δ*H*, and total Hx + HxO concentration) represent a set of traits that determine a model organism’s hypoxia tolerance and its variation with temperature. The well-documented trait variations in real animals (e.g., overall O_2_ supply capacity [26] and adaptation to hypoxia [27], gill surface area [28,29], and blood properties [30,31]) are simulated by scaling these parameters in the model. Our analysis aims to discern how such biological traits govern the shape of the resulting *P*_*crit*_ curves with respect to temperature.

Model simulations resemble standard closed system respirometry experiments used to determine *P*_*crit*_ values (Fig 2B) [32], in which O_2_ is depleted from the ambient water as it gets transferred to metabolizing tissues. Both the O_2_ concentrations and the fraction of O_2_-bound protein (HxO) decline in all compartments. Once O_2_ levels in the tissue compartment can no longer support resting metabolism, consumption slows down with the onset of hypoxemia, allowing *P*_*crit*_ to be diagnosed from the rate of environmental O_2_ depletion using breakpoint analysis (Materials and methods; full model in S1 Text).

Simulations across a range of temperatures yield the *P*_*crit*_ curve, which integrates the contribution of all traits to a single metric of hypoxia tolerance. Across a wide range of model parameters centered on the most common traits observed in marine organisms [7], the *P*_*crit*_ curves exhibit an overall rise with temperature, driven by the increase in metabolic rate. Both the chemical properties (Hx + HxO, Δ*H*, and *P*_50_) as well as the rate coefficients of supply and demand ($\hat{\alpha_{i}}$) have qualitative impacts on the *P*_*crit*_ curves that are intuitive. For example, a higher concentration of total oxygen transport protein (Hx + HxO) acts to lower the *P*_*crit*_ curve across all temperatures (Fig 2C), enhancing the tolerance to hypoxia. An equivalent effect can be obtained by increasing the biophysical supply coefficients, simulating changes such as a larger gill area or faster ventilation rate (**Fig 2D**).

The shape of the *P*_*crit*_ curves also exhibits a sensitivity to parameters that is quantitatively less intuitive, as the fractional change in *P*_*crit*_ is not always the same across the full temperature range (**Fig 2**). For example, a given increase in the ventilation rate does not lower the *P*_*crit*_ by the same fraction at all temperatures but instead has a larger impact under cold conditions than under warm conditions (Fig 2D). In other words, the *P*_*crit*_ curves resulting from a multistep supply chain can depart from simple exponential relationships with temperature, even when each single supply process accelerates exponentially with warming. We conclude that the well-known nonlinearities in blood–O_2_ binding are not the essential cause of this behavior, because the variation due to biophysical properties is similar to that induced by variations in blood chemistry and complex *P*_*crit*_ curves are observed in organisms without O_2_-binding proteins (e.g., *N*. *vectensis* in Fig 1; [17]). Instead, we focus our analysis on the mechanisms by which the linear combination of biophysical transfer processes in a multistep O_2_ supply chain leads to the complex patterns observed in *P*_*crit*_ curves.

**Fig 3:**
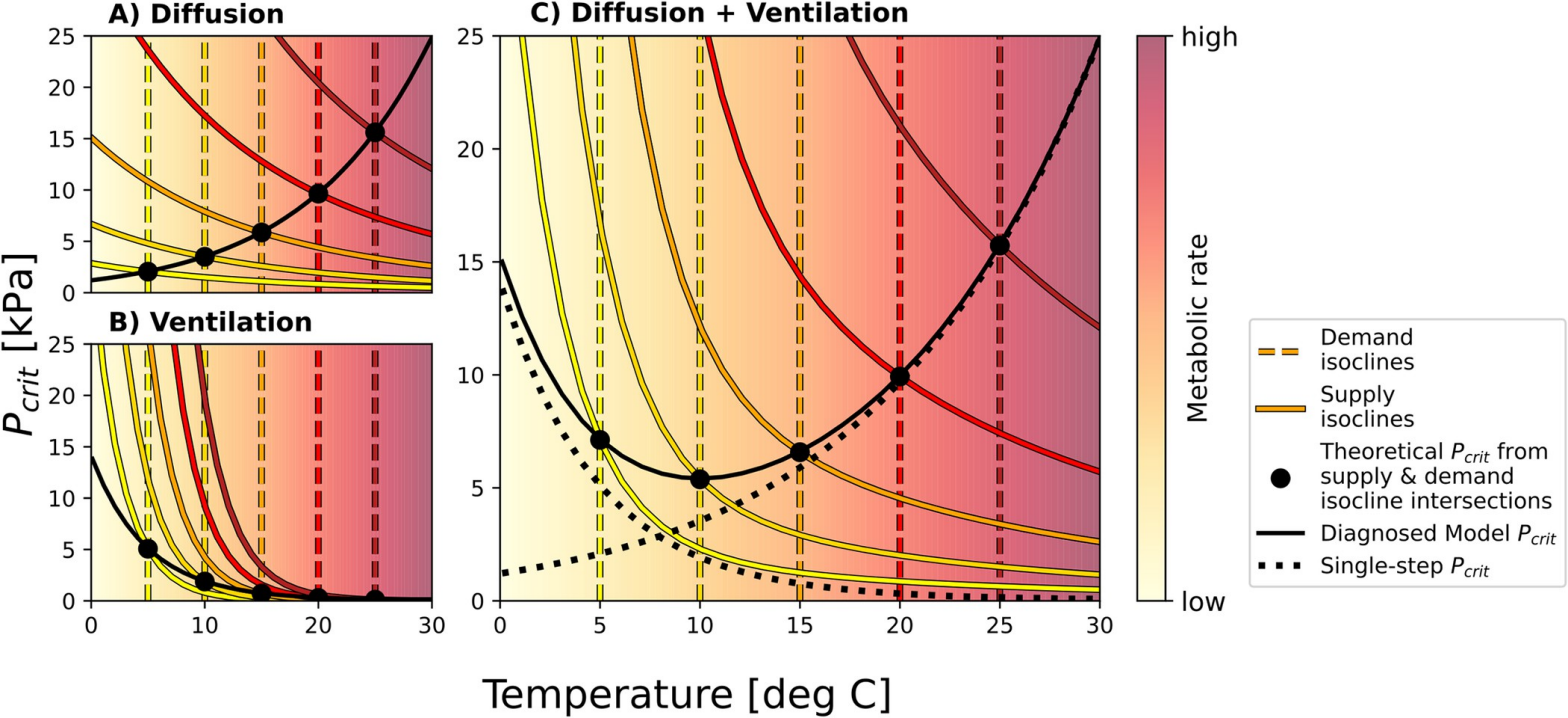
Modeled thermal optima in *P*_*crit*_ curves. At any given temperature, the model *P*_*crit*_ can be found analytically as the intersection (black dots) of the demand isocline (dashed colored) and the corresponding supply isocline (solid colored), aligning with the curve diagnosed from numerical simulations (solid black). **(A)** In a model with only diffusive gas exchange characterized by a smaller temperature sensitivity than metabolic demand, the isocline intersections yield an increasing *P*_*crit*_ curve. **(B)** Conversely, the steeper supply isoclines lead to a decreasing pattern in a single supply step model with ventilation that accelerates faster than metabolic demand. **(C)** Combining the 2 supply steps in series results in a *P*_*crit*_ curve that is the sum of the single step curves (dotted black), giving rise to a thermal optimum at intermediate temperatures. No experimental data underlie this figure. Code underlying this figure can be found in https://doi.org/10.6084/m9.figshare.24547255.

The origins of nonexponential *P*_*crit*_ curves can be demonstrated quantitatively in a model with a supply chain consisting only of ventilation and diffusive gas exchange (Fig **3**). In isolation, each step yields a simple (exponential) *P*_*crit*_ curve with a slope depending on the temperature sensitivities of supply and demand. The curve is increasing if metabolic demand accelerates faster with temperature than supply (shown for diffusion; Fig 3A), and decreasing if instead the temperature sensitivity of supply exceeds that of metabolism (ventilation; Fig 3B). Combining ventilation and diffusion in series results in a *P*_*crit*_ curve that is the sum of the 2 curves corresponding to the single steps and thus exhibits a minimum at an intermediate temperature (Fig 3C), similar to the observations.

The additive nature of the *P*_*crit*_ curve resulting from a linear supply chain can also be derived analytically from the system of model ODEs for more than 2 supply steps (S1 Text). Conceptually, this property can be thought of as analogous to an electrical circuit in which a fixed voltage is applied to a series of resistors. Just like the total voltage can be obtained as the sum of the individual voltage drops across each resistor, the total *P*_*crit*_ curve of a multistep supply chain can be obtained as the sum of the pO_2_ drops that drive each individual supply process.

A bowl-shaped *P*_*crit*_ curve can emerge if the supply chain includes processes that are both more and less sensitive to temperature changes than metabolism. In Fig 3C, the *P*_*crit*_ curve rises under warm conditions because a large pO_2_ gradient is required to drive sufficient diffusion at high temperatures. This is due to the fact that diffusion accelerates slower than metabolism with warming. On the other hand, the curve also remains flat or even reverses under cold conditions because a large pO_2_ gradient is required to provide sufficient O_2_ transport via ventilation at low temperatures, since this process has a higher temperature sensitivity than metabolism and thus slows down more strongly in cold water.

Because the critical pO_2_ differences required to drive the individual supply steps are not the same, the total *P*_*crit*_ curve is not equally sensitive to changes in the biologically controlled rate coefficients at all temperatures. In the example above, the change in *P*_*crit*_ at high temperatures due to a change in ventilation rate might be small or even negligible, while its response to a change in diffusivity might be substantial, even for the same relative increase in the biologically controlled parameter. More generally, a change in the coefficient of any supply process that accelerates faster with warming than metabolism will have the largest impact on *P*_*crit*_ under cold conditions, as in the case of ventilation. On the other hand, such an increase has the largest impact on *P*_*crit*_ under warm conditions for a supply process that accelerates slower than metabolism, such as diffusion. This relationship is particularly important for processes under immediate biological control like ventilation and circulation and has implications for understanding their temperature sensitivity. Incurring the energetic costs of accelerating heart rate or ventilation across the entire temperature range may not be beneficial if *P*_*crit*_ is instead much more sensitive to changes in diffusion at high temperatures. We illustrate this in a model variant with a ventilation rate that has a high temperature sensitivity at low temperatures but reaches an upper limit under warm conditions, as, for example, observed in [33–35]. The resulting change in *P*_*crit*_ at high temperatures compared to a simple exponential ventilation rate is minimal (Fig C in S1 Text). In this scenario, increasing the ventilation rate throughout the warm side of the temperature range barely impacts hypoxia tolerance, because O_2_ supply is largely determined by diffusion.

### Evidence from physiology

**Fig 4:**
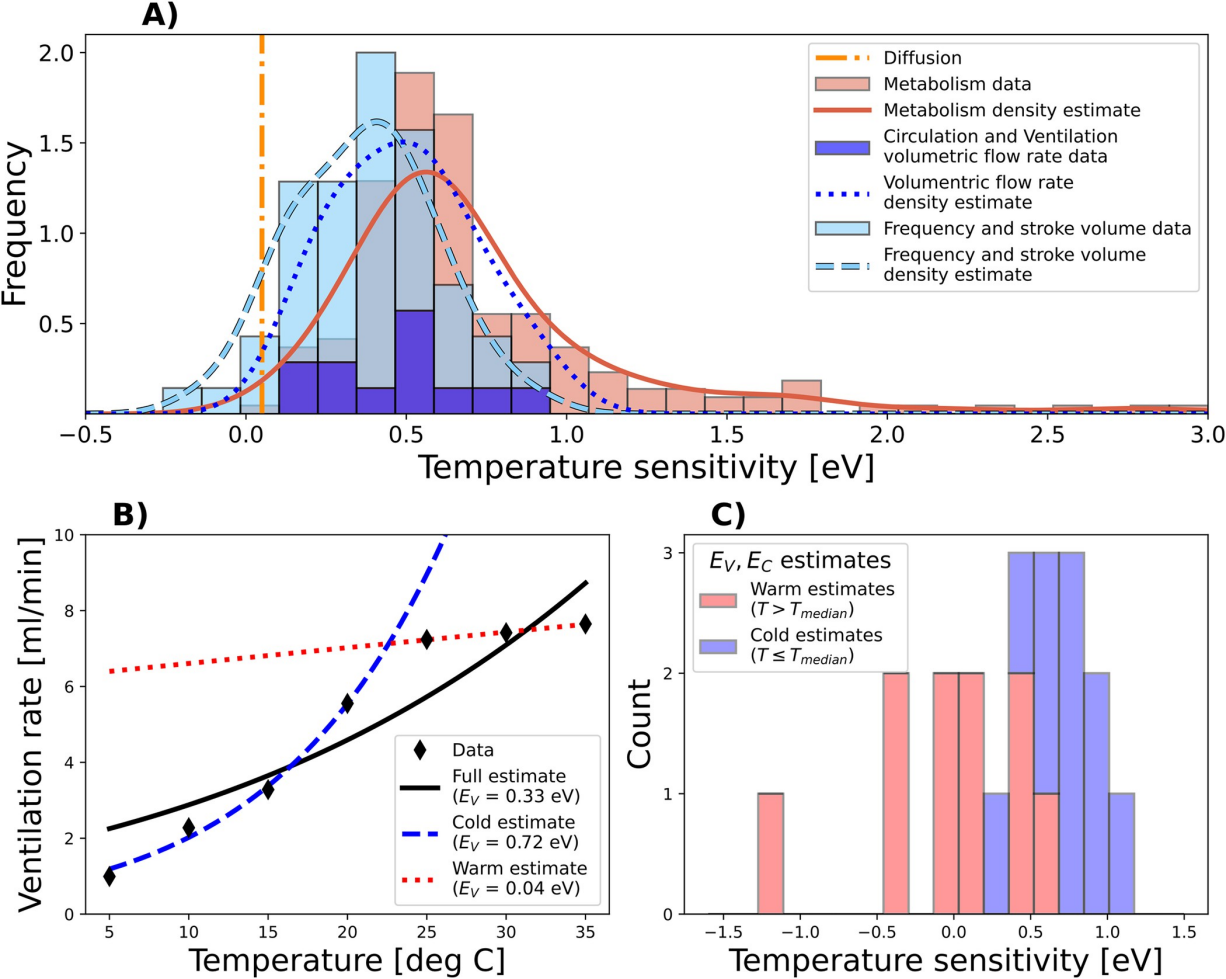
Temperature sensitivities of ventilation and circulation rates estimated from published experimental data. **(A)** The estimates (blue, *n* = 58 from 35 species) fall between the theoretical prediction for the sensitivity of diffusion (vertical orange line) and published estimates for the sensitivity of metabolic rates (*n* = 186 species; data from [7]) on average but with significant overlap, indicating the widespread potential for thermal optima in *P*_*crit*_. Lines show kernel density estimates of the trait frequency distributions. **(B)** Example for the estimation of *E*_*V*_ from published data across the full inhabited temperature range of the polychaete worm *Nereis succinea* (solid black; [33]) as well as under cold and warm conditions only (blue dashed and red dotted lines, respectively). **(C)** Estimated temperature sensitivities *E*_*V*_, *E*_*C*_ of volumetric ventilation and circulation rates from published data under cold (blue) and warm (red) conditions as illustrated in panel (**B**). The data underlying panels (**A**) and (**C**) can be found in S1 Data. The data underlying panel (**B**) can be found in [33].

To determine whether the physiological conditions for a thermal optimum in hypoxia tolerance are common among marine biota, we compiled experimental data on the temperature dependence of ventilation and circulation rates of aquatic water breathers (Materials and methods). The compilation covers 58 data sets from 35 species, including 21 chordates, 9 arthropods, 3 annelids, and 2 mollusks. Estimates of the temperature sensitivity parameters (*E*_*V*_, *E*_*C*_) that control the slope of the exponential relationship with temperature are obtained by fitting Arrhenius functions to the data. The results show an increase in ventilatory and circulatory activity with temperature in almost all species (i.e., *E*_*V*_, *E*_*C*_ > 0). The estimated sensitivities range from −0.14 eV to 0.9 eV and have a mean of 0.39 eV (±0.22 eV SD) that is much greater than the theoretical sensitivity of diffusion (*E*_*D*_ = 0.06 eV), which is the O_2_ supply process that is present in all organisms. Since these estimates include ventilation and circulation frequencies (e.g., heart rates) and stroke volumes (e.g., liters per heartbeat) in addition to actual volumetric flow rates (i.e., liters per min), they represent a lower bound on the sensitivity of (volumetric) ventilation and circulation rates as considered in the dynamic model. A higher sensitivity (0.49 eV ± 0.21 eV SD) is obtained if only volumetric rates (*n* = 12) are considered (Fig 4A).

The biophysical responses to temperature can be compared to existing estimates of the temperature sensitivity of metabolism (*E*_*M*_) with a mean of 0.71 eV [±0.46 eV SD] from a diverse set of 186 species [7]. If the traits of O_2_ supply and demand are considered independent, the estimated frequency distributions in Fig 4A predict that the conditions for thermal optima (i.e., *E*_*D*_ < *E*_*M*_ < *E*_*V*_, *E*_*C*_) are met in about 23% of species after accounting for the effect of decreasing solubility with temperature (S1 Text). However, this estimate likely represents a lower bound for the occurrence of bowl-shaped *P*_*crit*_ curves, for 2 reasons. First, supply sensitivities exceed that of demand in 7 of 17 species for which both estimates are available (S1 Data and Fig F in S1 Text). Thus, about 40% of species with paired E_V,C_ and E_M_ data meet this condition for having a thermal optimum in hypoxia tolerance.

Second, the thermal increase in ventilation and circulation rates is stronger on the cold side of the inhabited temperature range for many species (Fig 4B), where it is more likely to limit metabolism. We estimated the temperature sensitivity of volumetric flow rates in both warm and cold temperature ranges for all species with sufficient data (*n* = 10; Fig 4C). On the cold side, the mean *E*_*V*_ and *E*_*C*_ are 0.69 eV, significantly exceeding the warm side with a mean of 0.07 eV (*p* = 0.009). The difference remains significant if stroke volumes and frequencies are also considered (*n* = 40 from 25 species). Thus, the acceleration of ventilation and circulation rates slows down in the warmer part of the temperature range on average. This behavior is consistent with biophysical processes conferring little additional hypoxia tolerance at high temperatures for the associated energetic cost, with *P*_*crit*_ being most sensitive to diffusion under warm conditions, as illustrated in the model variant for ventilation (Fig C in S1 Text). It also means that interspecies estimates of *E*_*V*_, *E*_*C*_ for comparison to *E*_*M*_ (**Fig 4A**) are likely lower than they would be if measured at the cold edge, where these biophysical processes limit metabolism. Taken together, the interspecies distributions of *E*_*V*_, *E*_*C*_, and *E*_*M*_ (Fig 4A), and the higher temperature sensitivity of biophysical rates (circulation and ventilation) in colder waters (Fig 4B and 4C), including the reduction of these sensitivities in warmer waters, suggest that the conditions for thermal optima are commonly found among the traits of marine species.

### A Metabolic Index with thermal optima

We generalized the Metabolic Index introduced by Deutsch and colleagues [6] to account for the occurrence of complex shaped *P*_*crit*_ curves. The index is defined as the ratio of O_2_ supply to resting demand of an aquatic water breather in its environment, and it has been applied to understand how species biogeography is shaped by climate [6,10,11,18,36]. However, the original formulation assumed that *P*_*crit*_ varies exponentially with temperature. Our generalized version is able to reproduce the full range of behaviors exhibited by the dynamic model, requiring only 5 parameters, which can be calibrated from experimental data through a single equation.

The generalized Metabolic Index can be derived analytically from the dynamical model (S1 Text) but can also be developed heuristically from the electrical circuit analogy, noted above. The metabolic demand requires an equal rate of O_2_ flow (a “current”) to sustain it. Every step in the supply chain acts like a resistor with a biologically determined and temperature-dependent resistance whose inverse (a “conductance”) is given by the rate coefficient ${\hat{\alpha }}_{i}$ of the process. The flow at every step is the product of the conductance and the pO_2_ difference Δ*pO*_2_ (a “voltage”) between the compartments, which drives the current (Eq 1). Each step is associated with such a required voltage drop—a pO_2_ difference—determined by its single step conductance. The steps in the supply chain, from ventilation to diffusion and circulation, act as resistors wired in series, and the *P*_*crit*_ of the composite supply chain is the minimum “voltage” required to achieve an O_2_ supply matching demand and is the sum of the minimum pO_2_ differences of the single supply steps, as illustrated in **Fig 3C**.

The temperature-dependent rate coefficient (or “conductance”) ${\hat{\alpha }}_{i}$ of a single supply step can be expressed as $\alpha_{i}\cdot R(T,E_{S_{i}})$, where *α*_*i*_ denotes the value of the coefficient at reference temperature, which is scaled by an exponential (Arrhenius) function *R* with temperature sensitivity $E_{S_{i}}$ [eV]. More generally, in a chain with *n* supply steps in series, the total conductance of the chain is the reciprocal of the sum of single step resistances. When divided by metabolic demand *α*_*M*_·*R*(*T*, *E*_*M*_), the resulting expression for the generalized supply-to-demand ratio Φ is

$$\Phi =pO_{2}B^{\epsilon }{\left[\sum_{\left\{i=1\right\}}^{n}\frac{\alpha_{M}}{\alpha_{i}}R({T,E}_{i})\right]}^{-1}$$ (2)

where the *α*_*i*_ represent the supply rate coefficients at reference temperature, and $E_{i}=E_{M}-E_{S_{i}}$ [eV] denote the differences between the sensitivities of metabolic demand and the supply processes. The dependence of supply and demand on body mass *B* is reflected in the allometric exponent *ϵ* as in the original index [6].

The condition for the existence of a bowl-shaped *P*_*crit*_ curve, i.e., supply steps having temperature sensitivities both less than and greater than that of metabolic demand, thus reads $E_{S_{i}}<E_{M}<E_{S_{j}}$ for any 2 supply steps *i* and *j*. Eq 2 can include any number of supply processes. However, we find that *P*_*crit*_ curves generated by the full model (*n* = 3) can still be reproduced by curves assuming only 2 steps (Fig D in S1 Text). The generalized Metabolic Index in Eq 2 can reproduce *P*_*crit*_ curves that include the Hx/HxO system (Fig E in S1 Text), because the effects of the chemical blood component on the *P*_*crit*_ curve are qualitatively the same as those of the biophysical parameters (details in S1 Text). Adding more exponential curves also does not change the qualitative range of possible *P*_*crit*_ curves beyond those of concave-up bowl shapes (see Figs D and E in S1 Text). In such cases, the parameters can no longer be associated with single steps in the supply chain but instead capture the combined properties of the processes that limit the O_2_ supply toward the cold and warm ends of the temperature range, respectively. The asymmetry of the bowl-shaped curves can vary among species, depending on the values of the temperature sensitivities.

### Connecting physiology to biogeography

The Metabolic Index framework establishes a direct link between physiological traits and species distributions, as the range boundaries of a diverse set of species align more strongly with a lower threshold of O_2_ supply to demand predicted by the index than with either temperature or pO_2_ alone [7]. The generalized formulation has the potential to further improve this description of species habitats, especially at the cold edges of a species distribution.

To examine whether the thermal optima in physiological hypoxia tolerance are reflected in a species’ biogeography, we investigate state-space habitats of biogeographic occurrence data from the Ocean Biodiversity Information System [37] (see Materials and methods). For the species presented in Fig 1, the aerobic habitat conditions (T and pO_2_) are poorly represented in large-scale datasets (Fig G in S1 Text). However, for 2 additional species with physiological traits suggesting a thermal optimum, the starry flounder *Platichthys stellatus* and the shrimp *Oplophorus spinosus*, adequate occurrence and environmental data are available. In *Platichthys stellatus*, estimates from published experimental results yield a temperature sensitivity *E*_*M*_ = 0.68 eV for metabolism and *E*_*V*_ = 0.9 eV [38] for the ventilation rate, indicating a bowl-shaped O_2_ limitation. For *Oplophorus spinosus*, critical O_2_ pressures have been measured and display a minimum at intermediate temperatures [39], such that Eq 2 can be fit directly.

**Fig 5:**
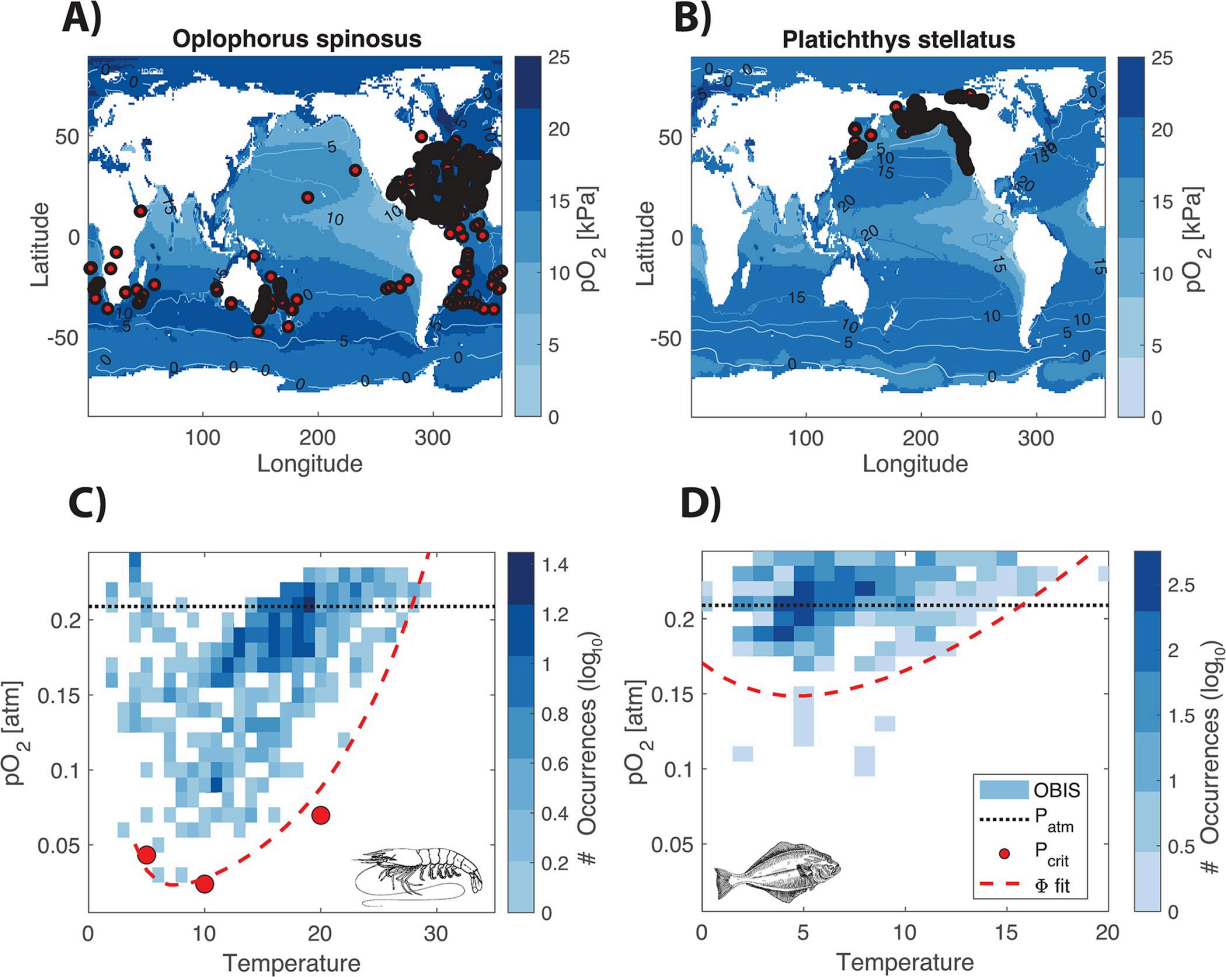
Biogeography and state-space habitats of species with thermal optima in hypoxia tolerance. Global occurrence data for 2 species, **(A)** the midwater shrimp *Oplophorus spinosus* and **(B)** the flounder *Platichthys stellatus*, from the Ocean Biodiversity Information System are mapped on the climatological temperature (lines) and pO_2_ (color field), from the World Ocean Atlas, averaged from the surface to the 95th percentile depth of reported species occurrences. The location (latitude, longitude, and depth) and month of each reported occurrence are matched to the climatological T and pO_2_, whose joint histogram is shown in the state-space habitat diagrams for each species. **(C)** In *Oplophorus spinosus*, measured *P*_*crit*_ values (red dots) as well as the curve fit based on the Metabolic Index (dashed red line) align with the lowest inhabited pO_2_ across the temperature range [10]. **(D)** In the flounder *Platichthys stellatus*, the *P*_*crit*_ curve (dashed red line) predicted from the Metabolic Index framework and physiological rates also exhibits a thermal optimum consistent with the occurrence data. The data underlying this figure can be found in https://doi.org/10.6084/m9.figshare.24547270. The code underlying this figure can be found in https://doi.org/10.6084/m9.figshare.24547255.

For both species, the environmental conditions in occupied habitats reveal a clear minimum in inhabited pO_2_ at intermediate temperatures, consistent with the measured and predicted physiological thresholds (Fig 5). In contrast, the minimum inhabited temperatures of each species are inconsistent with a model based on a lower threshold value of temperature that is independent of O_2_. Instead, minimum temperatures decrease to lower values as oxygen levels increase. Similar patterns are also observed in other species for which laboratory experiments on metabolic and O_2_ supply traits indicate thermal optima and for which sufficient occurrence data are available (details in S1 Text and Fig H in S1 Text; [18]).

In all these cases, the generalized Metabolic Index reveals how the reversal in hypoxia tolerance at low temperatures results from physiological traits, and how this bidirectionality is reflected in biogeographic ranges. In particular, it suggests that O_2_ limitation is the mechanism that restricts habitat toward the cold edges of species distributions.

## Discussion

The dynamic model of temperature-dependent hypoxia reveals that a series of biophysical O_2_ supply steps can give rise to thermal optima in hypoxia tolerance as observed in new high-resolution respirometry data. This occurs when the supply chain includes at least 2 processes such that one accelerates with temperature more slowly than metabolic demand, and another accelerates more rapidly. In this case, the process with a lower temperature sensitivity drives an increase in *P*_*crit*_ under warm conditions, while the more sensitive process leads to a reversal with higher *P*_*crit*_ in cold waters. A generalized Metabolic Index adequately captures these complex patterns in a single metric based on mechanistic principles.

Our analysis of available physiological evidence suggests that such bidirectional effects of temperature on hypoxia tolerance may not be uncommon in aquatic animals across taxonomic groups. Currently available estimates of the temperature sensitivity of ventilation and circulation rates in aquatic ectotherms imply the existence of thermal optima in a significant fraction of species because many estimates of *E*_*V*_/*E*_*C*_ exceed observations of *E*_*M*_, especially in cold waters where biophysical supply mechanisms appear most important. Sampling the involved traits across a broader range of the taxonomic, morphological, and ecological diversity is a key step toward further advancing and testing this framework and its implications, as there are only a few teleost and crustacean species for which all required physiological estimates are available. Our observation that the acceleration of ventilation and circulation rates slows down under warm conditions (Fig 4B and 4C and Fig C in S1 Text) also needs to be investigated more closely, as we only obtained 10 datasets that allowed this estimation. However, the causes and consequences of this phenomenon can also be investigated further theoretically by incorporating the metabolic cost incurred due to ventilation and circulation rates in the dynamic model. In particular, such a cost analysis should include the temperature-dependent viscosity of water and blood, which are not included here.

Our results highlight new opportunities to diagnose the role of O_2_ limitation in limiting the geographic range and body size of marine species. Patterns of body size under cold conditions in polar fish has recently been attributed to limitations on O_2_ supply [40]. The generalized Metabolic Index provides a framework to test this body size hypothesis, given additional measurements of *P*_*crit*_ across the range of body size and temperatures among these taxa [41]. In the environment, species minimum inhabited pO_2_ levels are elevated above physiological thresholds by the additional O_2_ requirements needed to sustain metabolic activity above the resting state, equivalent to the sustained metabolic scope [7,42], with the potential to influence T–pO_2_ relationships based on physiology alone. However, in case studies presented here, species’ biogeographic state space strongly reflected thermal optima in physiological hypoxia tolerance (Fig 5). In contrast to the sparsity of detailed physiological measurements, global occurrence data are available for a much larger number and diversity of marine species (e.g., in OBIS). Future work should aim to clarify and quantify such potentially synergistic physiological and ecological mechanisms driving the dependence of thermal tolerance on O_2_ supply and demand.

Oxygen limitation of aerobic metabolism at low temperature also has broad implications for marine ecosystems and their response to climate change [43]. Marine species richness is generally observed to decline toward the poles and equator from a peak in midlatitudes [44,45] and is often cited as being driven by gradients in ocean temperature, with coldest and warmest waters taken to inhibit diversity. Our results indicate that long-term aerobic energy constraints on viable habitat in cold water could be a physiological cause of this poleward diversity decline, just as aerobic constraints in warm water can explain the equatorial diversity dip [43]. At the same time, warming at species’ poleward range limits would relieve such aerobic constraints, allowing species to disperse toward, and establish in, higher latitudes. This mechanism could thus potentially explain widespread poleward range shifts of marine species seen in response to recent anthropogenic warming [45–47]. On longer timescales, O_2_ limitation at species’ cold edge habitat limits provides a novel mechanism for driving habitat loss and extinction risk during periods of global cooling [48,49].

## Materials and methods

### Ethics statement

The studied invertebrates were collected, reared, and handled according to all applicable permits and ethical standards of treatment. In particular, collection of the cephalopod *D*. *opalescencs* was carried out under California Department of Fish and Wildlife (CDFW) Scientific Collection Permit SC-13563, and husbandry and experiments were conducted under Institutional Animal Care and Use Committee (IACUC) #10643.

### Laboratory measurements

Critical O_2_ levels were measured following standard closed system respirometry protocols [17,50] for individuals of *T*. *tubifex* (*n* = 132), *N*. *vectensis* (*n* = 107), and *L*. *pictus* (*n* = 40). For the social squid *D*. *opalescens*, we measured critical O_2_ levels for 14 groups of 15 to 30 (median 20) animals following published closed system respirometry protocols for this species [51]. *P*_*crit*_ was determined by breakpoint analysis of the O_2_ draw down curve [52]. Full protocols are provided in S1 Text.

### Dynamic model

The pools and fluxes of O_2_ in a generic water breather are described by a nonlinear system of 8 ordinary differential equations. For each set of model parameters, simulations are performed across the temperature range from 0°C to 30°C until *P*_*crit*_ can be determined by breakpoint analysis from the rate of O_2_ draw down. All simulations were carried out in the Python language using the solve ivp function in Scipy [53] for numerical integration. The full model description is provided in S1 Text.

### Ventilation and circulation data

We compiled data on ventilation rates (*n* = 8), ventilation frequency (*n* = 18), ventilation stroke volumes (*n* = 6), circulation rates (*n* = 4), heart rates (*n* = 20), and heart stroke volumes (*n* = 2) of aquatic water breathers measured at 2 or more temperatures at atmospheric O_2_ levels. Estimates of the sensitivity parameters *E*_*V*_, *E*_*C*_ were obtained through least square fits of Arrhenius functions to the experimental data using the curve fit function and density estimates were obtained using the gaussian kde function in Scipy. A detailed description of the compiled data is provided in S1 Text, and all estimates are available in S1 Data.

### State-space habitats

State-space habitats were obtained by pairing species location data downloaded from the Ocean Biodiversity Information System [37] on June 6, 2022, with monthly temperature and O_2_ conditions from the World Ocean Atlas [54,55] according to the procedure described in [7]. All available state-space habitats are shown in Figs G and H in S1 Text. Additional information is also provided in S1 Text.

## Supporting information

S1 Text Supporting materials, methods, figures, and tables. File including supporting materials and methods, supporting figures A-H, supporting table A, and supporting references.
(PDF)

S1 Data Measured *P*_crit_ data and estimates of rate temperature sensitivities. Experimentally determined *P*_*crit*_ values and metabolic rates as well as estimates of the temperature sensitivity of ventilation, circulation, and metabolic rates of aquatic water breathers from published results.
(XLSX)
