# Supplementary material for: Thermal optima in the hypoxia tolerance of marine ectotherms: Physiological causes and biogeographic consequences
Source: PLoS Biol. 2024 Jan 16;22(1):e3002443. doi: 10.1371/journal.pbio.3002443 (PMC10790991; doi:10.1371/journal.pbio.3002443)
Supplement: S1 Text — File including supporting materials and methods, supporting figures A-H, supporting table A, and supporting references. (PDF) [file pbio.3002443.s001.pdf]

Supporting information:

Physiological causes and biogeographic consequences of thermal optima
in the hypoxia tolerance of marine ectotherms

Authors:

Martin-Georg A. Endress, Justin L. Penn, Thomas H. Boag, Benjamin P. Burford, Erik A. Sperling, Curtis A. Deutsch

Corresponding Authors: Curtis A. Deutsch, Martin-Georg A. Endress

Email: cdeutsch@princeton.edu, m.endress@uni-koeln.de

This supporting information includes:

Supporting materials and methods

Supporting figures A to H

Supporting table A

Supporting references

Other supporting materials for this manuscript include the following:

S1 Data. Experimentally determined Pcrit values and metabolic rates as well as estimates of the temperature sensitivity of ventilation, circulation and metabolic rates of aquatic water breathers from published results.

Supporting materials and methods

A. Respiration physiology materials and methods

Full P_{crit} data for all species are shown in Fig A.

Nematostella vectensis were lab reared and collected in 2017 at Stanford Hopkins Marine Station in Pacific Grove, California. *Lytechinus pictus* were purchased from the South Coast Bio-Marine Biological Supply in San Pedro, California, and analyzed in 2019. Collection of each species was seasonally timed so that the experimental temperature range was as close as possible to the current environmental temperature range. Upon arrival to the lab, animals were transferred into a synthetic seawater (33 practical salinity units, PSU) aquarium. Each animal was isolated, starved and acclimated to experimental temperature (± 0.3○C) for a minimum of 12 hours (up to 24 hours), then individually placed into a darkened respiration chamber with an oxygen optode attached to a Pyroscience FireSting 4-channel optical oxygen meter. Oxygen optodes were calibrated at each experimental temperature using a saturated sodium sulfite (Na2SO3) solution (30 g L-1) for 0% oxygen saturation, while a 100% oxygen saturation was achieved using seawater fully aerated with an air stone. A separate reservoir of synthetic seawater was used to fill experimental chambers prior to runs to ensure metabolites and microbes were absent prior to each run. Glassware was autoclaved between runs, and at temperatures above 25○C synthetic seawater was UV-treated and an antibiotic Streptomycin sulfate salt (C21H39N7O12· 1.5zH2SO4) solution (50 μmol L-1) was also added to further inhibit bacterial respiration. Bacterial respiration was negligible and was tracked using a blank respiration chamber that was filled with identically treated synthetic seawater. At the end of experiments specimens were blotted dry and weighed to obtain wet mass. Standard closed system respirometry protocols were used to measure P_{crit}. The oxygen content of each chamber was continuously recorded at one second intervals using Pyro Oxygen Logger software, allowing P_{crit} to be determined with break-point analyses on the oxygen draw down curve [1]. This is possible because P_{crit} coincides with the onset of anaerobiosis, representing the switch from oxyregulatory aerobic respiration independent of pO2, to oxyconformity, whereby the animal

slows respiration in response to decreasing pO2. This is reflected in a breakpoint between slopes representing oxygen consumption (standard metabolic rate), and drastically reduced oxyconformation oxygen consumption as anaerobic metabolism makes up an increasingly larger fraction of ATP turnover. To determine the effect of temperature on the metabolic rate and hypoxia tolerance, experiments were repeated across five temperatures which span natural environmental range. A minimum of 10 individuals were measured for each temperature to account for natural variation, with 107 *Nematostella vectensis* and 40 *Lytechinus pictus* individuals measured in total across all temperatures. Resting M_{O_2} at different temperatures was determined from the slope of the oxygen drawdown curve in each experimental chamber prior to P_{crit} and incorporating the adjusted volume of water, mass of the organism, and time elapsed using the following equation [2]:

$$M_{O_2} = \frac{\frac{[O_2]_2-[O_2]_1}{t_2-t_1} \cdot 3600 \cdot V}{B}$$

where

M_{O_2} = mass-normalized metabolic rate (μmol L^{-1} g^{-1} h^{-1})

$[O_2]$ = oxygen concentration (μmol L^{-1})

t = time (s)

V = water volume (L)

B = body mass (g)

To determine standard metabolic rate at each temperature, recorded MO2 measurements were resampled 10,000 times to produce a frequency distribution. The mean of the lowest 10% of points was used while all MO2 measurements above this were assumed to be associated with spontaneous activity [3]. We used closed-system respirometry to measure the temperature dependence of P_{crit} in groups of *D. opalescens*. Experiments controlled for other factors known to influence resting

and routine levels of metabolic demand of marine ectotherms: body size, digestion, and oxygen availability [4]. Adult *D. opalescens* were captured in nearshore waters of Monterey Bay, CA, USA from April-May 2018 using small barbless jigs and then transported in aerated seawater to an indoor aquarium facility at Hopkins Marine Station, Pacific Grove, CA. Only undamaged squid caught by the sucker cups on the arms were retained for this study. No injuries were observed in any animals used in experiments (n = 309; 187 male, 111 female). Squid mass ranged from 17.7-89.1 g, with an average of 41.4 g. Squid were housed together in groups in a 3200 L circular tank with filtered flow-through seawater (20 L min-1) at ambient temperature (12-14○C) for 24-48 h during which they received one meal of live minnows (*Pimephales promelas*) per 24 h period. Group respiration was accomplished using a custom-built respirometer. The system is composed of two 600 L circular tanks arranged side-by-side a buffer tank for temperature control and an insulated test tank for metabolic measurements (484 L test section when sealed with a transparent lid). The test tank was visually shielded with opaque plastic sheeting suspended over a fence encircling the circumference, but squid could be observed through a small opening. All experiments occurred at the same time of day and under the same lighting conditions. After introduction to the test tank, groups of squid were allowed to acclimate to experimental conditions for 12 h with flow-through seawater (range = 12.5-13.2○C, mean = 13○C). This resulted in at least 24 h without food before measurement began. We used a HQ40D multimeter and LDO101 oxygen sensor (Hach Co., USA) to record oxygen concentration, oxygen saturation, and temperature at 0.1 Hz in the test tank. During the temperature ramp, an immersion heater and immersion chiller (Process Technology, USA) connected to the buffer tank respectively increased or decreased the water temperature at 1 ○C h-1 as measured in the test tank. Once the temperature ramp was complete, measurement began and continued until squid started showing obvious signs of fatigue, or until oxygen concentrations reached 0.5 mgL-1(measurement duration range = 6.4-23.9 h, median = 11.9 h). Measurements were conducted at a suite of temperatures within the ecologically relevant range that *D. opalescens* typically encounters in the CCS. Three groups were measured at each of the following treatments: 7.5, 10, 13, 16, and 19○C. Due to a failure in power supply to the multimeter, data from one

experiment at 16○C were not recorded and thus there was a total of n = 14 groups. The average temperature during measurement was never more than 0.4○C from the target temperature. The routine activity *D. opalescens* displayed during measurements, where squid used a mixture of fin undulation and jet propulsion to slowly move head- and tail-first and to hold station, sufficiently mixed water within the test tank. Using functions in the respR package in R [5], we applied the segmented regression approach [1] to determine the breakpoint of oxygen consumption curves and calculate P_{crit}, the oxygen partial pressure below which a stable rate of oxygen uptake at routine levels was not maintained [6]. Oxygen consumption rates were consistent from experiment start until P_{crit} was reached. Control experiments containing no squid were conducted to quantify microbial background respiration. No background respiration was detected over experimentally relevant durations; we therefore did not apply any correction to group rates for background respiration.

B. Dynamic model formulation

The time-dependent transfer of O_2 from the environment to the metabolizing tissues is simulated using a system of 8 ODEs representing the concentrations of dissolved O_2 (X) as well as the free (F) and bound (B) forms of O_2 transporting proteins in the four model compartments as shown in Fig. B.

The full ODE system reads:

$$\text{Dissolved } O_2\ X\ :\begin{cases} V_W \frac{d}{dt} X_W &= -\hat{\alpha}_V (X_W - X_B) \\ V_B \frac{d}{dt} X_B &= \hat{\alpha}_V (x_W - X_B) - \hat{\alpha}_D \frac{(X_B - X_G)}{\kappa_H} \\ V_G \frac{d}{dt} X_G &= \hat{\alpha}_D \frac{(X_B - X_G)}{\kappa_H} - \hat{\alpha}_C (X_G - X_I) + k_b B_G - k_f X_G^{n_H} F_G \\ V_I \frac{d}{dt} X_I &= \hat{\alpha}_C (X_G - X_I) + k_b B_I - k_f X_I^{n_H} F_I - \hat{\alpha}_M \end{cases} \tag{1-4}$$

$$\text{Free transport protein } F\ :\begin{cases} V_G \frac{d}{dt} F_G &= -\hat{\alpha}_C (F_G - F_I) + k_b B_G - k_f X_G^{n_H} F_G \\ V_I \frac{d}{dt} F_I &= \hat{\alpha}_C (F_G - F_I) + k_b B_I - k_f X_I^{n_H} F_I \end{cases} \tag{5-6}$$

$$O_2 - \text{bound transport protein } B\ :\begin{cases} V_G \frac{d}{dt} B_G &= -\hat{\alpha}_C (B_G - B_I) - k_b B_G + k_f X_G^{n_H} F_G \\ V_I \frac{d}{dt} B_I &= \hat{\alpha}_C (B_G - B_I) - k_b B_I + k_f X_I^{n_H} F_I \end{cases} \tag{7-8}$$

Here X_W, X_B, X_G, X_I denote the concentrations of dissolved O_2 $[\frac{\mu mol}{l}]$ in the external water, external boundary layer, internal gill and internal tissue compartments with corresponding volumes V_W, V_B, V_G, V_I $[l]$, while F_G, F_I and B_G, B_I denote the concentrations of free and O2-bound transport proteins $[\frac{\mu mol}{l}]$ in the gill and tissue compartments, respectively. The transport of O_2 depends on temperature $T[K]$ and is realized through ventilation $\hat{\alpha}_V(T)[\frac{l}{min}]$ between the environmental water and the boundary layer, through diffusive gas exchange $\hat{\alpha}_D(T)[\frac{\mu mol}{min\ kpa}]$ between the boundary layer and the gill volume and through circulation $\hat{\alpha}_C(T)[\frac{l}{min}]$ between the gill volume and the tissues. For diffusive gas exchange, the concentration difference between the compartments is converted to a partial pressure difference with the temperature-dependent solubility $\kappa_H(T)$ $[\frac{\mu mol}{l\ kpa}]$ of O_2 in seawater, calculated using coefficients from [7] for [8] for a salinity of 35 PSU, assuming

that the solubility of O_2 is not significantly different in the body fluid/blood for the purposes of this model (while O_2 solubility is lower in body fluids than in seawater, e.g. [9], any temperature-independent effect only changes the rate constant of diffusion). Finally, O_2 is consumed in the tissue compartment through metabolism $\hat{\alpha}_M$ $[\frac{\mu mol}{min}]$.

These supply and demand rates $\hat{\alpha}_V, \hat{\alpha}_D, \hat{\alpha}_C, \hat{\alpha}_M$ are defined as

$$\begin{aligned} \hat{\alpha}_V(T) =& \alpha_V \cdot R(T, E_V) \\ \hat{\alpha}_D(T) =& \alpha_D \cdot R(T, E_D) \\ \hat{\alpha}_C(T) =& \alpha_C \cdot R(T, E_C) \\ \hat{\alpha}_M(T) =& \alpha_M \cdot R(T, E_M) \end{aligned} \tag{9-12}$$

with constants α_x and dimensionless temperature dependent factors taking the form of Arrhenius functions

$$R(T, E_x) = \exp\left(\frac{-E_x}{k_B}(\frac{1}{T} - \frac{1}{T_{ref}})\right) \tag{13}$$

with temperature sensitivities E_x $[eV]$. Throughout the paper, we use the reference temperature $T_{ref} = 288.15$ $[^\circ K] \equiv 15$ $[^\circ C]$ and k_B denotes the Boltzmann constant. The temperature-independent α_x represent the supply and demand fluxes at the reference temperature determined by biological traits like resting metabolic rate, gill surface area, heart rate etc. These rate coefficients and temperature sensitivities are varied throughout the investigation to explore their impacts on hypoxia tolerance. For diffusion, we estimate $E_D \approx 0.21$ [eV] by fitting an Arrhenius function to relative diffusivity (κ) values

$$\frac{\kappa(T)}{\kappa(T_{ref})} = \frac{T}{T_{ref}} \frac{\mu(T_{ref})}{\mu(T)} \tag{14}$$

with the dynamic viscosity of seawater $\mu(T)$ calculated according to [10] for a salinity of 35 PSU. After accounting for temperature-dependent solubility κ_H, this yields a value of $E_D \approx 0.06$ eV.

Typical values of the biophysical parameters and values used for the compartment volumes are provided in Table A (Model variables and parameters).

The model also contains a blood component with free (F) and bound (B) forms of transport protein circulating between the gill and the tissue compartments according to $\hat{\alpha}_C(T)$. These bind or release O_2 with forward and backward reaction rates k_f $[\frac{l^3}{\mu mol^2\ min}]$, k_b $[\frac{l}{min}]$, respectively, with a dissociation curve following Hill's equation where the O_2 pressure producing half-saturation, denoted P_{50}, is temperature-dependent [11]. The fraction Y of protein bound to O_2 is

$$Y = \frac{[B]}{[B] + [F]} = \frac{pO_2^{n_H}}{P_{50}^{n_H} + pO_2^{n_H}} \tag{15}$$

where

$$P_{50}(T) = P_{50}^{T_{ref}} \cdot \exp\left(\frac{\Delta H}{R_{Gas}}(\frac{1}{T} - \frac{1}{T_{ref}})\right) \tag{16}$$

with the half-saturation pressure at the reference temperature $P_{50}^{T_{ref}}$, the Hill coefficient n_H and the enthalpy of the binding reaction ΔH, and R_{Gas} is the gas constant. The O_2 concentration K_{50} producing half saturation ('microscopic dissociation constant') can be converted from the pressure $K_{50} = \kappa_H P_{50}$. Since the ratio of the reaction rates k_f, k_b determining the equilibrium is more relevant than the absolute values of the rates for our purposes, we fix the forward rate to be much faster than diffusive gas exchange and compute k_b as $k_b = k_f K_{50}^{n_H}$ from the equilibrium constant $K_{eq} = \frac{k_b}{k_f} = K_{50}^{n_H}$.
The values used for these chemical parameters are shown in Table A (Model variables and parameters).

At each temperature, we integrate the ODE system using the *solve_ivp* solver in Python [12]. We start each simulation with a concentration of dissolved O_2 typical of surface seawater of 300

$\frac{\mu mol}{l}$ in all compartments and with corresponding equilibrium concentrations of the transport protein. After an initial spin-up time of 50 min with constant $X_W(t)$ to ensure that the saturated O_2 concentration can support the model metabolic rate, $X_W(t)$ starts to evolve according to SI Eqn. 1. To determine P_{crit}, we use the 'broken stick' method [13] by applying a segmented linear regression following [1] to the rate of change of $X_W(t)$. We use the time t_{crit} at which the O_2 concentration in the tissue first drops below a critical limit near zero ($X_I(t_{crit}) \leq 0.05\ \frac{\mu mol}{l}$) as an initial guess for the single breakpoint and restrict the Regression to the interval $[50, t_{crit} + \frac{t_{crit}}{5}]$. If X_I never drops below this threshold during the default simulation time of 1000 min, the time is doubled and the process is repeated until a P_{crit} value is obtained.

To illustrate the diminishing effects of supply processes that accelerate faster than metabolism under warm condition, we formulate a simple model variant with a modified ventilation rate with a slowed acceleration above 20 ° C:

$$\hat{\alpha}_V = \begin{cases} \alpha_V R(T, E_V) & T < 288.15K \\ \frac{2\alpha_V}{1+\exp(-\lambda(T-T_{ref})} & T \geq 288.1515K \end{cases} \tag{17}$$

where $\lambda = \frac{2E_V}{k_B T_{ref}^2}$ is chosen to match the value and slope of the Arrhenius ventilation at T_{ref}. The modified rate as well as the resulting change in P_{crit} is shown in Fig. C.

C. Metabolic Index derivation

The generalized Metabolic Index (Eqn. 2 in main text) can be derived from the dynamical model without the blood proteins F, B in SI equations 1-4. To determine P_{crit} analytically, we consider the case of a very large environmental volume $V_W \rightarrow \infty$, which is equivalent to a setting with a constant environmental O_2 level $X_W(t) = X_W$ that is not depleted over time. Intuitively, this represents the fact that respiration of a small model organism does not significantly change the ambient O_2 level in the ocean or a large tank. Under this assumption, we can find the constant concentration X_W^{crit} that

results in a steady state O_2 level of zero in the tissue, $X_I^{ss} = 0$, which corresponds to the criterion for reaching P_{crit} in our model (see B Dynamical model formulation). From SI Equation 4, we have

$$\frac{d}{dt}X_I = \hat{\alpha}_C(X_G^{ss} - 0) - \hat{\alpha}_M = 0$$
$$\Rightarrow X_G^{ss} = \frac{\hat{\alpha}_M}{\hat{\alpha}_C}.$$

Substituting this in SI Eqn 3 yields

$$\frac{d}{dt}X_G = \hat{\alpha}_D \frac{(X_B^{ss} - \frac{\hat{\alpha}_M}{\hat{\alpha}_C})}{\kappa_H} - \hat{\alpha}_M = 0$$
$$\Rightarrow X_B^{ss} = \frac{\hat{\alpha}_M \kappa_H}{\hat{\alpha}_D} + \frac{\hat{\alpha}_M}{\hat{\alpha}_C}.$$

Finally, inserting X_B^{ss} and X_G^{ss} in SI Eqn. 2 yields

$$\frac{d}{dt}X_B = \hat{\alpha}_V(X_W^{crit} - \frac{\hat{\alpha}_M \kappa_H}{\hat{\alpha}_D} - \frac{\hat{\alpha}_M}{\hat{\alpha}_C}) - \hat{\alpha}_M = 0$$
$$\Rightarrow X_W^{crit} = \frac{\hat{\alpha}_M}{\hat{\alpha}_V} + \frac{\hat{\alpha}_M \kappa_H}{\hat{\alpha}_D} + \frac{\hat{\alpha}_M}{\hat{\alpha}_C}.$$

Converting to partial pressure results in the expression for P_{crit}

$$P_{crit} = \frac{\hat{\alpha}_M}{\hat{\alpha}_V \kappa_H} + \frac{\hat{\alpha}_M}{\hat{\alpha}_D} + \frac{\hat{\alpha}_M}{\hat{\alpha}_C \kappa_H} \quad (18)$$

This corresponds to the MI

$$\Phi = pO_2 \left(\frac{\hat{\alpha}_M}{\hat{\alpha}_V \kappa_H} + \frac{\hat{\alpha}_M}{\hat{\alpha}_D} + \frac{\hat{\alpha}_M}{\hat{\alpha}_C \kappa_H} \right)^{-1} \quad (19)$$

which matches Eqn. 2 in the main text after substituting SI Eqns. 9-12. On the other hand, the same result is obtained when considering the ratio of O_2 supply to demand directly, since the total

(potential) supply S_{tot} of the supply chain is

$$S_{tot} = pO_2 \cdot \hat{\alpha}_{tot}$$

with the total conductance of the linear chain

$$\hat{\alpha}_{tot} = \left(\sum_{i=1}^{n} \frac{1}{\hat{\alpha}_i} \right)^{-1}.$$

Now the ratio $\frac{S_{tot}}{\hat{\alpha}_M}$ once again yields SI Eqn. 19.

While the full system including the transport protein concentrations F, B does not yield a simple expression for P_{crit} and Φ, simulated P_{crit} curves can be adequately fit based on Eqn. 2 in the main text with only $n = 2$ supply steps, as shown in Fig. D and E for a range of biophysical and chemical parameters. In particular, the figures illustrates the fact that the presence of O_2 transporting proteins essentially enhances the supply capacity of circulation in the model, with changes in the total concentration of protein achieving a similar effect to changes in the parameter α_C (compare circulation column in Fig D with panel A in Fig E) and changes in the enthalpy of the binding reaction corresponding to changes in the sensitivity E_C (compare circulation column in Fig D with panel D in Fig E).

D. Ventilation and circulation data

We compiled data on the thermal sensitivity of ventilation rates (n=8), ventilation frequencies (n=18), ventilation stroke volumes (n=6), circulation rates (n=4), heart rates (n=20) and heart stroke volumes (n=2) in aquatic water breathers at atmospheric O2 levels measured at 2 or more temperatures from the literature. For each of the 58 datasets from 35 species (25 marine, 10 freshwater), the temperature dependence of the measured quantity was estimated by fitting an Arrhenius equation $k \cdot R(T, E)$ from SI Eqn. (13) to the data normalized to the lowest experimental

temperature and estimating the sensitivity parameter E. Least-Square Fits were performed using the *scipy.optimize.curve fit* function. The results are provided in S1 Data.

The temperature sensitivities of ventilation (32 estimates) and circulation (26 estimates) were not significantly different in terms of means according to a F-test (p = 0.1608), which was performed after testing for equal variances (Levene-test, p = 0.8103) and checking normality (Shapiro-Wilk-test, p = 0.5071 and p = 0.5816, respectively). Therefore, the ventilation and circulation estimates were combined for further analysis.

We estimated the expected frequency of the supply temperature sensitivities E_V, E_C of volumetric flow rates exceeding the sensitivity of metabolism E_M. To that end, we estimated the trait frequency distributions of these sensitivities through kernel density estimation using the gaussian kde function in Scipy and sampled from the resulting densities (n = 100000). To account for the effect of decreasing solubility κ_H with temperature as it appears in SI Eqn. (18), the density of ventilation and circulation rates was shifted by the temperature sensitivity of solubility ($\approx$ 0.144 eV from [8]). This procedure yields an expected frequency for $E_V, E_C \geq E_M$ of 23 %, which corresponds to the expected fraction of species with thermal optima in hypoxia tolerance if the traits are considered independent. On the other hand, in the 17 species for which both estimates are available, we find that $E_V, E_C \geq E_M$ in 7 cases (frequency $\approx$ 41 %) after accounting for solubility effects, indicating that the traits determining the thermal sensitivity of O_2 supply and demand are unsurprisingly not independent in real organisms. The temperature sensitivities in these 17 species are comparable to estimates obtained from all species (demand 0.58 ± 0.15 eV, supply 0.43 eV ± 0.21 eV, see Fig F).

To evaluate whether E_V, E_C are themselves temperature dependent, we estimated the parame-ter separately above and below the median temperature in the 40 datasets from 25 species with 4 or more distinct experimental temperatures. Estimates were again obtained by fitting Arrhenius functions $k \cdot R(T, E)$ to the data. For the 10 estimates of volumetric flow rates, the cold side mean of 0.69 eV far exceeds the warm side mean of 0.07 eV (paired sample t-test, p = 0.009). The difference remains significant when frequencies and stroke volumes are also considered (cold side

mean 0.43 eV, warm side mean of 0.15 eV, paired sample t-test, p = 0.0085). This is illustrated Fig 4C in the main text. If the analysis is restricted to the 24 datasets from 16 species with temperature ranges of at least 5○C both above and below the median experimental temperature, the difference between the cold side mean (0.48 eV) and the warm side mean (0.22 eV) remains similar. The average experimental temperature range is 17.95○C (10.95○C cold side, 7.51○C warm side).

E. State-space projections

The curves in Fig 5 were obtained from Eqn. (2) with n = 2 supply steps while accounting for any physiological evidence available for the respective species.

For *Oplophorus spinosus*, Eqn. (2) was fit directly to the experimental P_{crit} data [14] while constraining the temperature sensitivities to $0 \leq E_1 \leq 0.8$ [eV] and $-0.7 \leq E_2 \leq 0$ [eV] respectively, based on the estimate $E_M \approx 0.7308$ eV estimated in [15] from the data of [14].

For *Platichthys stellatus*, no estimates of P_{crit} are available, so the curve was tuned to $E_1 = 0.65$ eV eV and $E_2 = -0.25$ eV based on the available estimates of $E(V) \approx 0.9$ eV and $E_M \approx 0.68$, as well as the theoretical value of $E_D \approx 0.06$ eV for diffusion.

In addition to the state-space habitats shown in Fig 5, we also provide the habitats for the 4 species with experimental P_{crit} data (Fig 1) in Fig G. There are only very few occurrences available in OBIS for three of the species (*L. pictus, N. vectensis, T. tubifex*), two of which are intertidal (*N. vectensis*) or inhabit freshwater and brackish habitat (*T. tubifex*). In these cases, the use of monthly mean values for temperature and O_2 conditions from the World Ocean Atlas likely does not reflect the range of environmental conditions these species experience in their natural habitats, such as strong variations in O_2 frequently experienced by intertidal animals [16], and limits the interpretation of the state-space projections. While there is a larger number of occurrences for *D. opalescens* (see panel A in Fig G), the gradients in temperature and O_2 these squid experience during offshore migrations and nearshore spawning activity [17] are likely also not well represented in monthly climatologies.

Finally, we provide the state-space habitats for the 8 marine species for which both estimates of the

supply and demand temperature sensitivities are available (Fig H, S1 Data). The state-space habitats reveal bowl-shaped patterns in the two additional marine species for which physiological evidence suggests the presence of thermal optima (*C. crangon, S. solea*). Such bowl-shaped patterns are also present in the state-space habitats of some of the remaining species, for which the available estimates only explain the warm edge (e.g. *G. morhua*, see panel E) in Fig H). However, there is no species with all required sensitivity estimates for metabolic demand, ventilation rate and circulation rate to test the emergence of state-space habitats without thermal optima.

Table A. Model variables and parameters. Descriptions and default values of state variables and parameters in the dynamic model.

Symbol	Description	Default	Unit
T	Temperature	273.15 - 303.15	K
k_B	Boltzmann's constant	8.6173e-5	eV / K
R_{Gas}	Gas constant	0.008314	kJ / K / mol
T_{ref}	Reference temperature	288.15	K
$\kappa_H(T)$	Solubility of O_2 in seawater	SI Ref. 7	μmol / liter / kPa
μ	Dynamic viscosity of seawater	SI Ref. 10	kg / min / s
X_W	O_2 concentration in environment	300	μmol / liter
X_B	O_2 concentration in boundary layer	300	μmol / liter
X_G	O_2 concentration in gill	300	μmol / liter
X_I	O_2 concentration in tissue	300	μmol / liter
F_G	Free protein concentration in gill	$1 - Y$	mmol / liter
F_I	Free protein concentration in tissue	$1 - Y$	mmol / liter
B_G	Bound protein concentration in gill	Y	mmol / liter
B_I	Bound protein concentration in tissue	Y	mmol / liter
V_W	Volume of environmental compartement	20	liter
V_B	Volume of boundary layer compartement	0.005	liter
V_G	Volume of gill compartement	0.005	liter
V_I	Volume of tissue compartement	0.005	liter
α_V	Ventilation rate at T_{ref}	0.002	liter / min
α_D	Diffusion rate at T_{ref}	0.029	μmol / min / kPa
α_C	Circulation rate at T_{ref}	0.00025	liter / min
α_M	(Resting) metabolic rate at T_{ref}	0.05	μmol / liter / min
E_V	Temperature sensitivity of ventilation	0.8	eV
E_D	Temperature sensitivity of diffusion	0.21	eV
E_C	Temperature sensitivity of circulation	0.8	eV
E_M	Temperature sensitivity of metabolism	0.5	eV
$P_{50}^{T_{ref}}$	Half-saturation pressure at T_{ref}	3	kPa
Y	Fraction of bound O2-transport protein	SI Eqn. 15	-
n_H	Hill coefficient	2	-
ΔH	Enthalpy of O2 binding reaction	-30	kJ / mol
K_{eq}	Equilibrium constant of O2 binding reaction	$\frac{1}{(P_{50}\kappa_H)^{n_H}}$	μmol^2 / liter^2
k_f	Forward/O2 binding reaction rate	$10\alpha_D$	liter^3/ μmol^2 / min
k_b	Backward/O2 dissociation reaction rate	$k_f K_{eq}$	liter / min

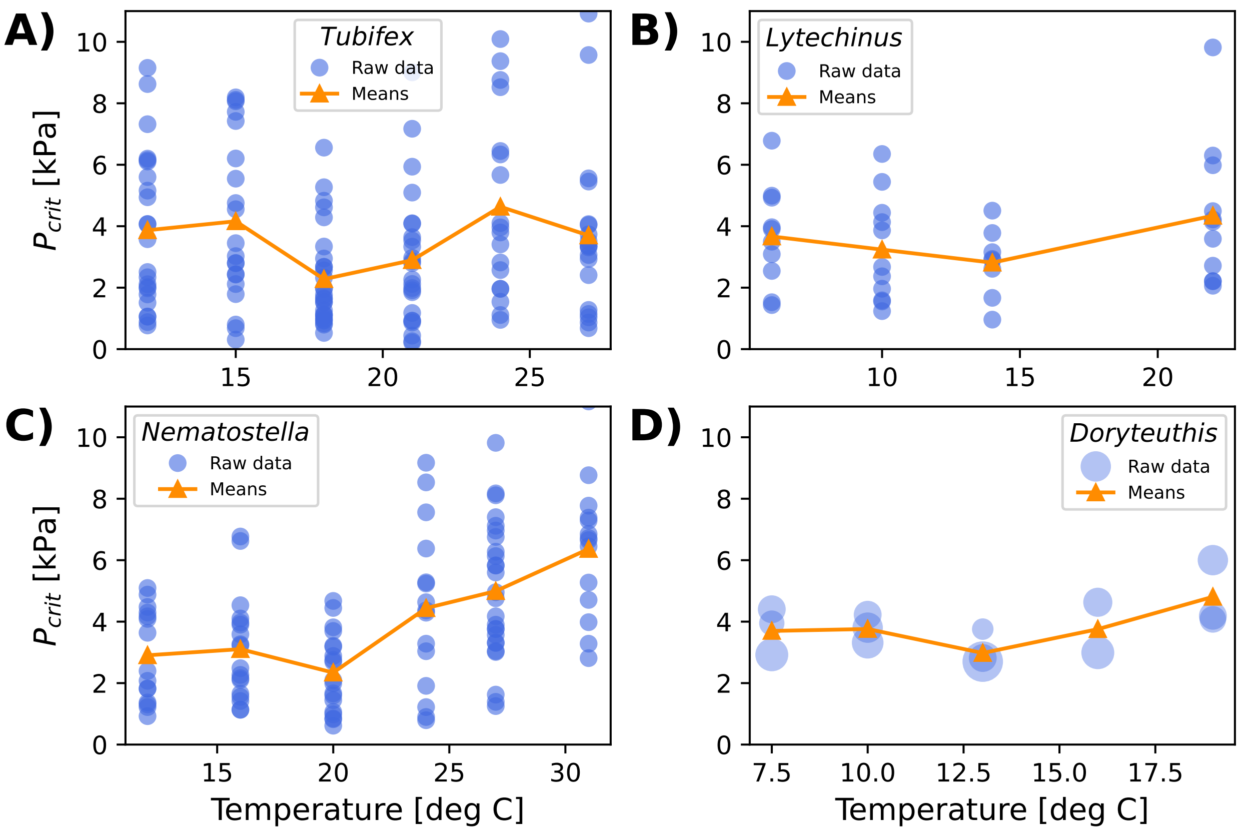

Fig. A Raw P_{crit} measurements (blue) and mean values as in Fig 1 (orange) for **A)** the oligochaete worm *Tubifex tubifex*, **B)** the sea urchin *Lytechinus pictus*, **C)** the anemone *Nematostella vectensis* and **D)** the market squid *Doryteuthis opalescens*. Experimental protocols and the procedure for determining P_{crit} values are provided in section A of supporting materials and methods (respiration physiology experiments). Data underlying this figure can be found in S1 Data.

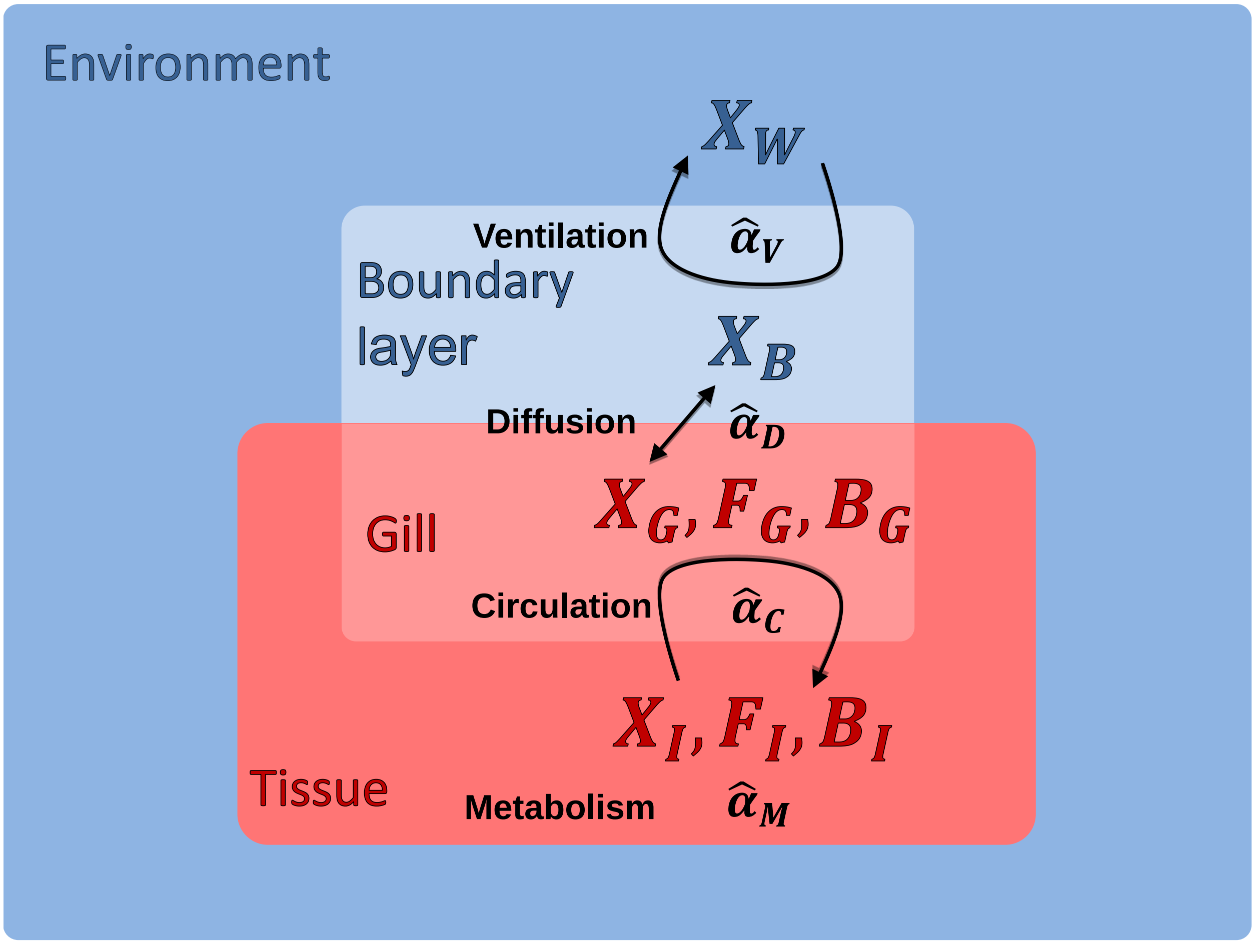

Fig. B Illustration of state variables for dissolved O2 (X), free (F) and bound (B) O2-transport protein as well as temperature-dependent O2 supply and demand processes ($\hat{\alpha}$) used in the dynamical ODE model. No experimental data underlie this figure.

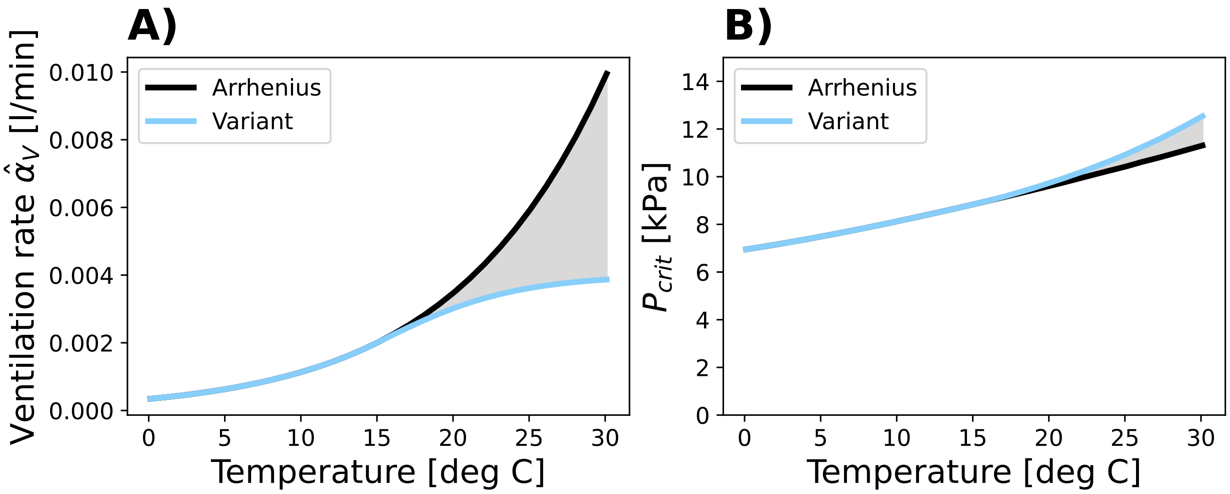

Fig. C Variant of the dynamical model featuring a ventilation rate with slower acceleration at high temperatures. **A)** Above 20 ○C, the modified ventilation rate following SI Eqn. (17) accelerates significantly slower than the default rate governed by the exponential (Arrhenius) relationship in SI Eqn. (9). **B)** The resulting change in P_{crit} at high temperatures is minimal because ventilation is characterized by a larger temperature sensitivity than metabolism (E_V = 0.8 eV, E_M = 0.5 eV). No experimental data underlie this figure. Code underlying this figure can be found in https://doi.org/10.6084/m9.figshare.24547255.

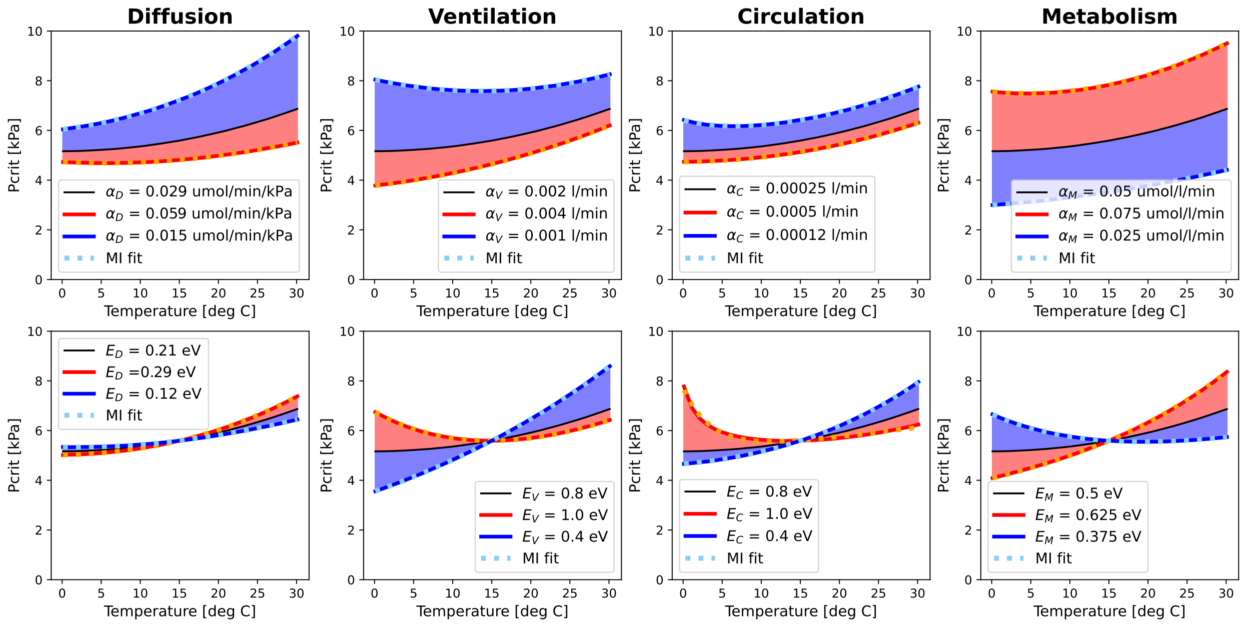

Fig. D Sensitivity analysis of the model for deviations of the biophysical parameters (coefficients α in the top row, temperature sensitivities E in the bottom row) from their default values given in Table A. In all cases, fits based on Eqn. (2) from the main text with 2 supply steps (dashed) capture the simulated P_{crit} curves accurately. No experimental data underlie this figure. Code underlying this figure can be found in https://doi.org/10.6084/m9.figshare.24547255.

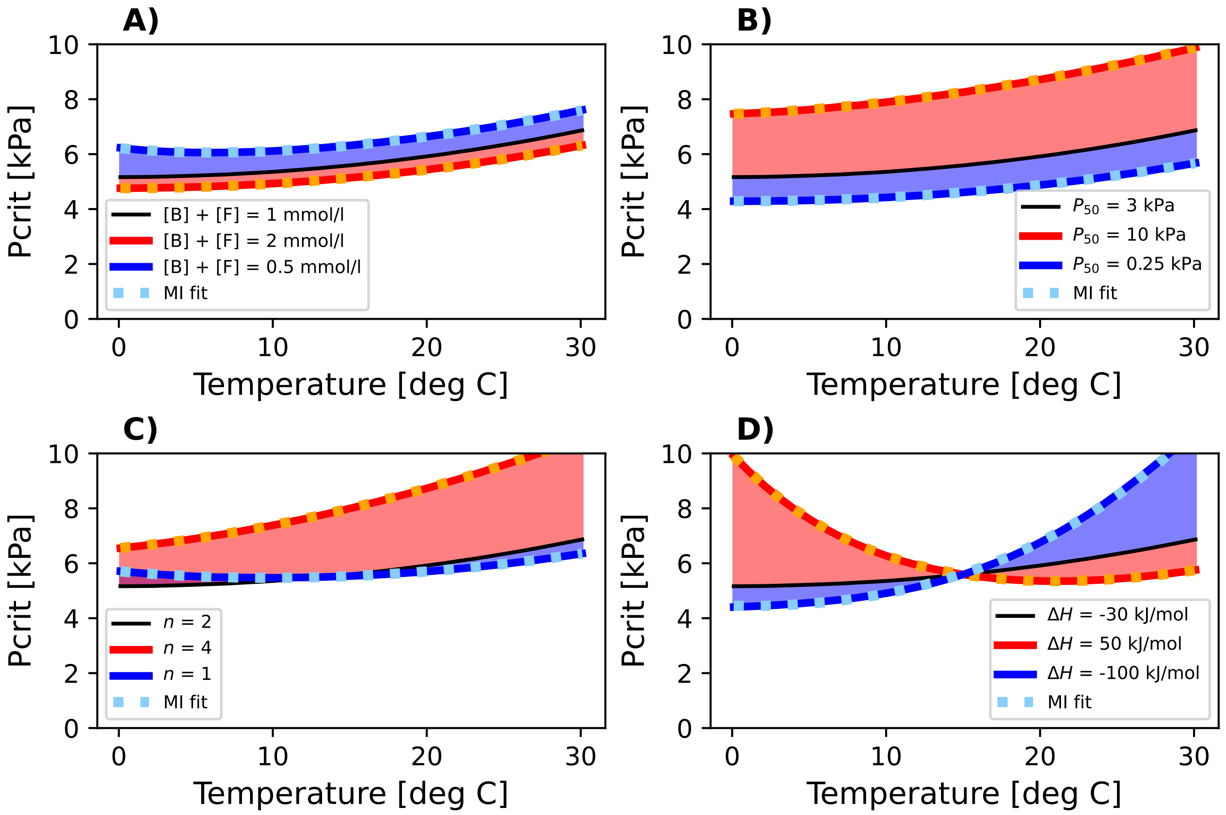

Fig. E Sensitivity analysis of the model for deviations of the parameters characterizing the oxygen transport protein system from their default values given in Table A. **A)** Total protein concentration Hx+HxO **B)** Reference half-saturation pressure $P_{50}^{T_{ref}}$ **C)** Hill coefficient n_H **D)** Enthalpy of the oxygen binding reaction ΔH. In all cases, fits based on Eqn. (2) from the main text with 2 supply steps (dashed) capture the simulated P_{crit} curves accurately. No experimental data underlie this figure. Code underlying this figure can be found in https://doi.org/10.6084/m9.figshare.24547255.

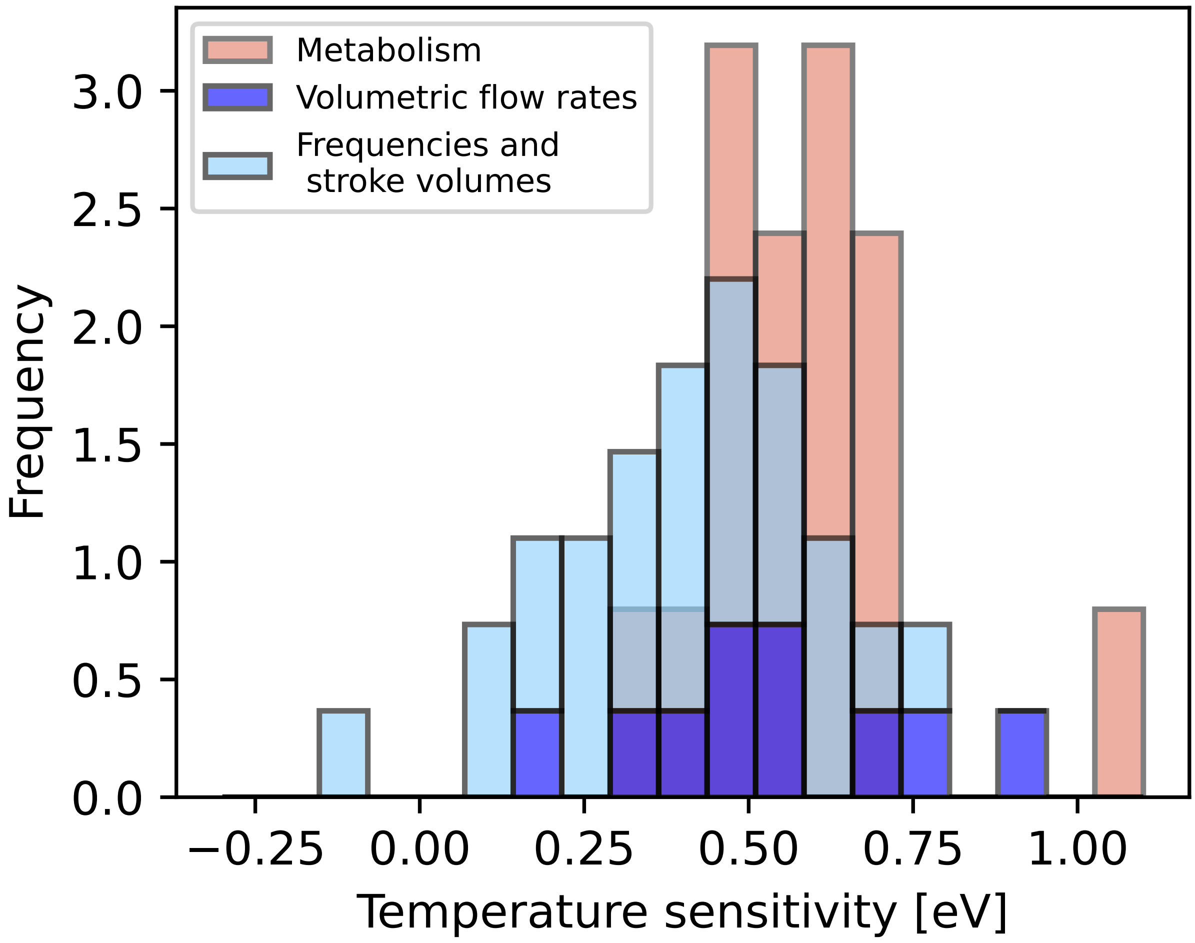

Fig. F The sensitivity estimates for ventilation and circulation as well as metabolic demand in the 17 species for which both quantities are available yield a distribution that is comparable to that obtained from all species (Fig 4A). Data underlying this figure can be found in S1 Data.

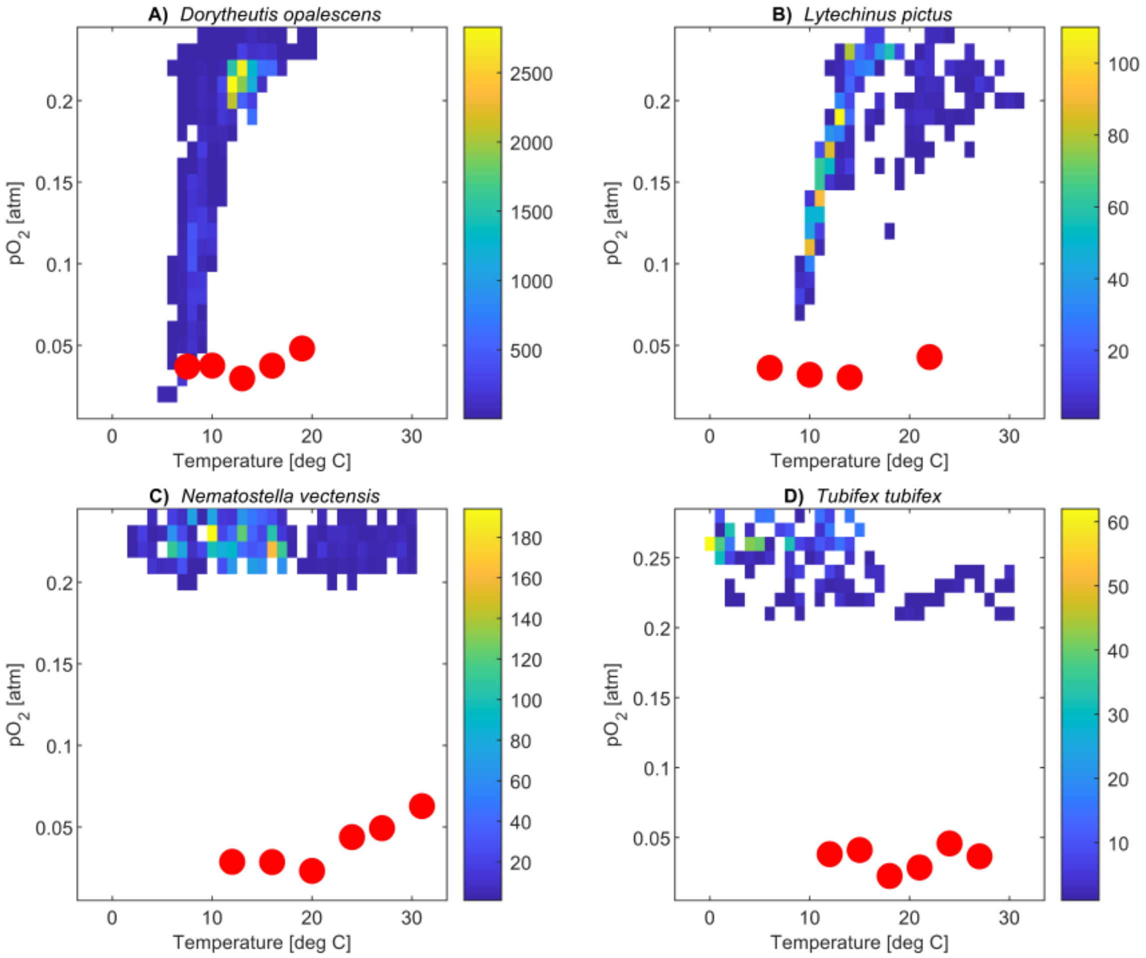

Fig. G State-space habitats 303 of OBIS occurrences along with mean experimental P_{crit} values for the 4 species presented in Fig 1, including **A)** market squid from the California Current System, **B)** an outer shelf sea urchin, **C)** an intertidal anemone and **D)** a sludge worm that mostly inhabits freshwater habitat. Occurrence data underlying this figure can be found in https://doi.org/10.6084/m9.figshare.24547270. Pcrit data underlying this figure can be found in S1 Data. Code underlying this figure can be found in https://doi.org/10.6084/m9.figshare.24547255.

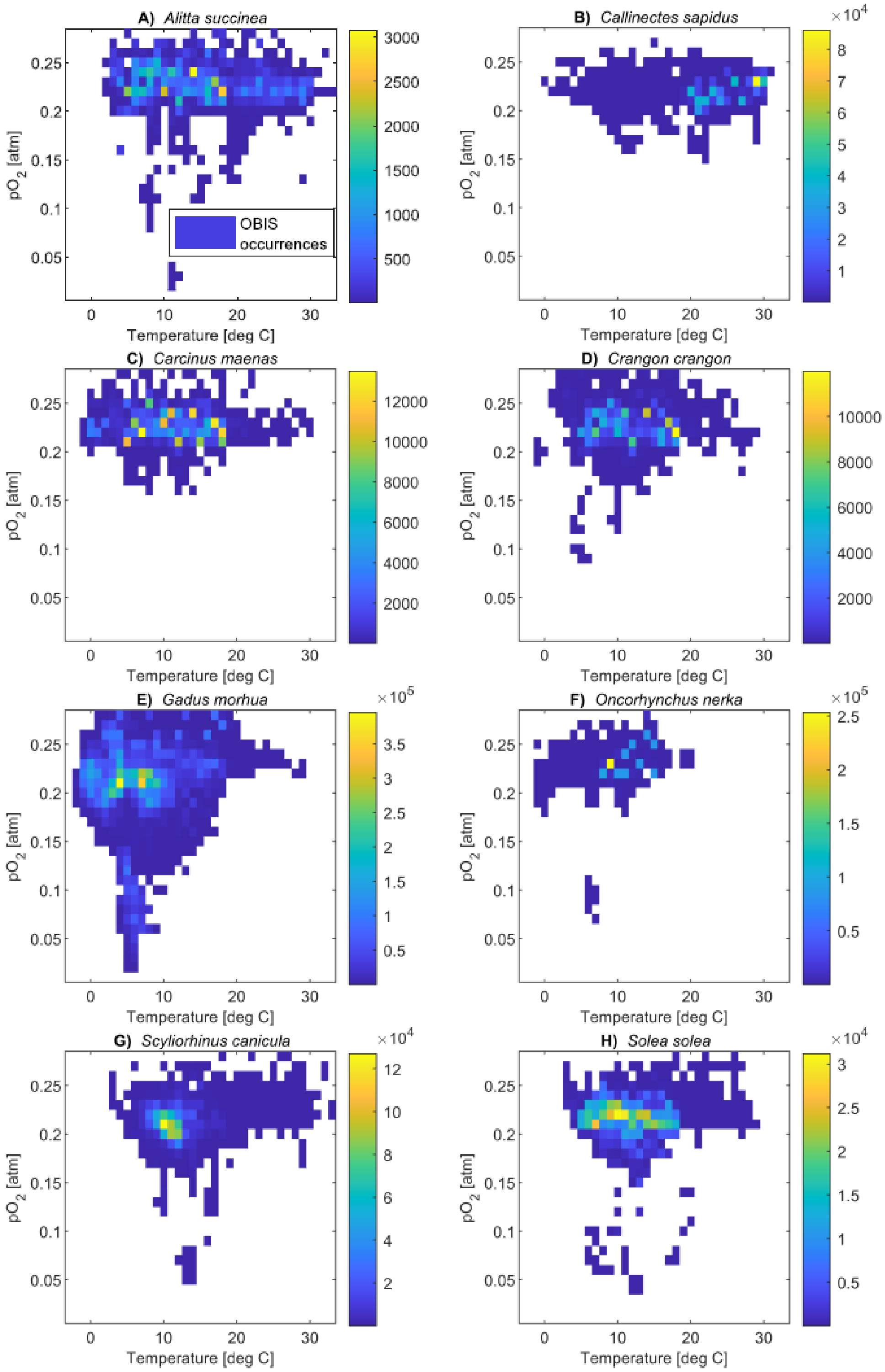

Fig. H State-space habitats for all species with sufficient physiological data on temperature dependent O2 supply and demand rates as well as occurrence data in OBIS. Data underlying this figure can be found in https://doi.org/10.6084/m9.figshare.24547270. Code underlying this figure can be found in https://doi.org/10.6084/m9.figshare.24547255.

SI References

[1] Muggeo VMR. segmented: an R Package to Fit Regression Models with Broken-Line Relationships. R News. 2008;8(1):20–25.

[2] Steffensen JF. Some errors in respirometry of aquatic breathers: How to avoid and correct for them. Fish Physiology and Biochemistry. 1989;6(1):49–59. doi:10.1007/BF02995809.

[3] Clark TD, Sandblom E, Jutfelt F. Aerobic scope measurements of fishes in an era of climate change: respirometry, relevance and recommendations. Journal of Experimental Biology. 2013;216(15):2771–2782. doi:10.1242/jeb.084251.

[4] Burford B, Carey N, William G, Goldbogen J. Grouping reduces the metabolic demand of a social squid. Marine Ecology Progress Series. 2019;612. doi:10.3354/meps12880.

[5] Harianto J, Carey N, Byrne M. respR - An R package for the manipulation and analysis of respirometry data. Methods in Ecology and Evolution. 2019;doi:10.1111/2041-210X.13162.

[6] Rogers N, Urbina M, Reardon E, McKenzie D, Wilson R. A new analysis of hypoxia tolerance in fishes using a database of critical oxygen level (P crit). Conservation Physiology. 2016;4:cow012. doi:10.1093/conphys/cow012.

[7] Garcia HE, Gordon LI. Oxygen solubility in seawater: Better fitting equations. Limnol Oceanogr. 1992;37(6):1307–1312. doi:10.4319/lo.1992.37.6.1307.

[8] Benson BB, Krause D. The concentration and isotopic fractionation of oxygen dissolved in freshwater and seawater in equilibrium with the atmosphere1: Oxygen solubility in seawater. Limnol Oceanogr. 1984;29(3):620–632. doi:10.4319/lo.1984.29.3.0620.

[9] Lallier F, Truchot JP. Hemolymph oxygen transport during environmental hypoxia in the shore crab, Carcinus maenas. Respiration Physiology. 1989;77(3):323–336. doi:https://doi.org/10.1016/0034-5687(89)90120-5.

[10] Sharqawy MH, Lienhard JH, Zubair SM. Thermophysical properties of seawater: a review of existing correlations and data. Desalination and Water Treatment. 2010;16(1-3):354–380. doi:10.5004/dwt.2010.1079.

[11] Wells R. Chapter 6 Blood-Gas Transport and Hemoglobin Function: Adaptations for Functional and Environmental Hypoxia. Fish Physiology. 2009;27:255–299. doi:10.1016/S1546-5098(08)00006-X.

[12] Virtanen P, Gommers R, Oliphant TE, Haberland M, Reddy T, Cournapeau D, et al. SciPy 1.0: Fundamental Algorithms for Scientific Computing in Python. Nature Methods. 2020;17:261–272. doi:10.1038/s41592-019-0686-2.

[13] Reemeyer JE, Rees BB. Standardizing the determination and interpretation of P_{crit} in fishes. J Exp Biol. 2019;222(18):jeb210633. doi:10.1242/jeb.210633.

[14] Cowles DL, Childress JJ, Wells ME. Metabolic rates of midwater crustaceans as a function of depth of occurrence off the Hawaiian Islands: Food availability as a selective factor? Mar Biol. 1991;110(1):75–83. doi:10.1007/bf01313094.

[15] Deutsch C, Penn JL, Seibel B. Metabolic trait diversity shapes marine biogeography. Nature. 2020;585(7826):557–562. doi:10.1038/s41586-020-2721-y.

[16] Mcmahon B. Physiological Responses to Oxygen Depletion in Intertidal Animals. Integrative and Comparative Biology - INTEGR COMP BIOL. 1988;28:39–53. doi:10.1093/icb/28.1.39.

[17] Zeidberg L. In: Advances in Squid Biology, Ecology and Fisheries. Part I – Myopsid Squids; 2013. p. 159–204.
